# A Complete Fossil-Calibrated Phylogeny of Seed Plant Families as a Tool for Comparative Analyses: Testing the ‘Time for Speciation’ Hypothesis

**DOI:** 10.1371/journal.pone.0162907

**Published:** 2016-10-05

**Authors:** Liam W. Harris, T. Jonathan Davies

**Affiliations:** 1 Department of Biology, McGill University, 1205 Docteur-Penfield Avenue, Montreal, Quebec, Canada, H3A 1B1; 2 African Centre for DNA Barcoding, University of Johannesburg, PO Box 524, Auckland Park, 2006, Johannesburg, South Africa; University of Sydney, AUSTRALIA

## Abstract

Explaining the uneven distribution of species richness across the branches of the tree of life has been a major challenge for evolutionary biologists. Advances in phylogenetic reconstruction, allowing the generation of large, well-sampled, phylogenetic trees have provided an opportunity to contrast competing hypotheses. Here, we present a new time-calibrated phylogeny of seed plant families using Bayesian methods and 26 fossil calibrations. While there are various published phylogenetic trees for plants which have a greater density of species sampling, we are still a long way from generating a complete phylogeny for all ~300,000+ plants. Our phylogeny samples all seed plant families and is a useful tool for comparative analyses. We use this new phylogenetic hypothesis to contrast two alternative explanations for differences in species richness among higher taxa: time for speciation versus ecological limits. We calculated net diversification rate for each clade in the phylogeny and assessed the relationship between clade age and species richness. We then fit models of speciation and extinction to individual branches in the tree to identify major rate-shifts. Our data suggest that the majority of lineages are diversifying very slowly while a few lineages, distributed throughout the tree, are diversifying rapidly. Diversification is unrelated to clade age, no matter the age range of the clades being examined, contrary to both the assumption of an unbounded lineage increase through time, and the paradigm of fixed ecological limits. These findings are consistent with the idea that ecology plays a role in diversification, but rather than imposing a fixed limit, it may have variable effects on per lineage diversification rates through time.

## Introduction

It is now well recognised that species richness is unevenly distributed across the tree of life and the origin of this variation is the subject of significant debate [1–5]. In seed plants, variation in species richness between sister clades is often large, leading to highly imbalanced phylogenetic topologies [6, 7]. Despite the long history of research on these patterns and an increasingly accurate understanding of the evolutionary history for many taxonomic groups, the mechanisms underlying differences in species richness remain largely undetermined, with possible explanations spanning biological, historical, geographical and neutral processes [8]. A better understanding of the causes of imbalance in species richness across lineages could provide insight into the mechanisms governing the evolution and proliferation of life. Here, we reconstruct a complete phylogenetic tree for all seed plant families, and use this tree to evaluate two alternative explanations for variation in species richness among clades: ‘time for speciation’ versus ‘ecological limits’ [4].

The time for speciation effect [4], whereby older clades, having had more time to diversify, are represented by a larger number of species, implicitly assumes that diversification is unbounded. Under this model, we should therefore expect to see a positive relationship between clade age and the logarithm of species richness, regardless of the range of clade ages [4]. Further, simple calculations of net diversification rate (r) should be able to explain a significant proportion of the variation observed in seed plant diversity. In contrast, the ecological limits model assumes that external factors (ecological limits) restrict clade expansion. According to this alternative model, young clades, which have yet to reach their ecological limits, may show a positive correlation with clade age, but as clades get older and species richness approaches the ecological limit for the clade, the relationship will be lost [9]. In addition, a clade that has reached saturation in species number would appear to have a faster diversification rate when observed at some point in the past than it does now (time [*t*] is increasing while species number [*S*] remains constant) despite the fact that its diversification rate during its initial expansion does not change [9].

Large, well sampled phylogenies for species-rich groups allow us to examine variation in evolutionary rates across clades and through time (e.g. amphibians [10], birds [11], mammals [12,13], plants [7,14–17]), and thus provide an opportunity for evaluating macroevolutionary models of diversification. However, the reconstruction of complete, dated, phylogenetic trees for large groups has remained a challenge, and is confounded by poor and uneven sampling of taxa. With advances in molecular sequencing technologies following the development of the polymerase chain reaction (PCR) in the 1980’s, there has been a rapid accumulation of phylogenetic data, but the collection of these data has been largely piecemeal. For some species or clades we have sequences for multiple genes, or even whole genomes, whereas other branches on the tree of life are only sparsely represented (see e.g. [18]). To overcome this data shortfall various heuristics have been developed to assemble large phylogenetic trees [19]. Supertree approaches combine many small but overlapping phylogenies to form a single, more inclusive phylogeny [20,21]. An alternative approach for assembling large phylogenies is to use expert opinion. Phylogenetic hypotheses are constructed using best available knowledge, typically assuming a fixed backbone tree representing the taxonomic relationships among major lineages and including missing taxa as polytomies, and, when possible, manually resolving relationships based on independent phylogenetic hypotheses (see e.g. [22]). In flowering plants, where regional richness might sum to many thousands of species, such approaches are common-place and have been automated in the Phylomatic online tool [23].

Meta-phylogeny reconstruction methods, such as those described above, have obvious utilitarian value, but they also have a number of limitations. Critically, branch length data are typically absent or poorly estimated, confounding studies of evolutionary rates, either in diversification or character evolution. Although it is possible to estimate branching times from topology alone, such approaches must assume, *a priori*, a particular model of diversification (e.g. [13,24]); hence, estimating evolutionary rates on such trees can be circular (see [25]). When molecular sequence data are available, branch lengths may be estimated directly (e.g. see [7]), and it is possible to derive branch lengths for different genes on different parts of the tree and then calibrate on common nodes if there is sufficient taxonomic overlap in gene coverage and genetic evolution is clock-like [26].

Here, we generate a complete and robustly dated phylogenetic tree for all seed plant families by combining current knowledge on plant family relationships with sequence data on four gene regions with high taxonomic coverage (chloroplast genes *rbcL*, *matK* and *atpB*, and the nuclear ribosomal RNA-encoding gene *18S*) and 26 fossil constraints. Our approach complements efforts towards the generation of the comprehensive tree of life [27], but differs in that we additionally provide branch length estimates, a critical parameter for generating and testing phylogenetic hypotheses on evolutionary rates. We use this tree topology and data on the species richness of plant families to map patterns of clade diversification across the phylogeny and contrast predictions of the ‘time for speciation’ versus ‘ecological limits’ models of clade expansion.

## Methods

### Phylogeny Reconstruction

Phylogeny reconstruction followed a three-step procedure. First, a backbone topology was constructed following the Angiosperm Phylogeny Group III (APG III) classification [28], and missing families placed using best available information. Second, molecular branch lengths were optimized on to the family-level backbone using RAxML v.8.0.0 [29] and four gene regions (*rbcL*, *matK*, *atpB* and *18S rRNA*) mined from BOLD (http://www.barcodinglife.com/) and GenBank (http://www.ncbi.nlm.nih.gov/genbank/). Third, the tree was made ultrametric and branch lengths calibrated to millions years using Bayesian analysis in BEAST v.1.8.0 [30]. We did not attempt to reconstruct the phylogeny directly from the raw sequence data because our aim here was not to generate a new hypothesis of seed plant evolutionary relationships, but to take advantage of existing knowledge based on multiple studies using separate lines of evidence, and summarized by experts (i.e. APG III). While current computational tools allow us to rapidly generate phylogenetic hypotheses for many hundreds of taxa, we do not believe such an estimate here would improve on published work that has targeted specific clades and gene regions to maximally resolve phylogenetic relationships (see e.g. [31] for a similar approach to that used here).

The Phylomatic supertree [23] of all plants was downloaded and trimmed to generate a cladogram of all seeded plant families. This topology represents an expert summary of current higher level angiosperm molecular systematics following the APG III [28,32]. We note that, following the initial tree reconstruction described here, APG IV has now been released [33]; however, we retained the APG III backbone so as to be consistent with the taxonomy used to extract family species richness data (see below). This cladogram was enforced as a topological constraint in all subsequent analyses. Three families (Apodanthaceae, Cynomoriaceae and Vahliaceae) were not represented on the megatree and were added manually based on the best available data. Apodanthaceae was added as a polytomy with Anisophyllaceae and Cucurbitaceae [34], Cynomoriaceae was added as a sister to Rosaceae [35], and Vahliaceae was added as a polytomy with lamiids [28].

DNA sequence data were obtained for all 425 seeded plant families accepted by the Plant List v.1.1 (http://www.theplantlist.org/), which is informed by APGIII [28], and the 2001 World Checklist of Conifers [36], to generate a molecular matrix with one sequence per family for each of four genes, *rbcL*, *matK*, *atpB* and *18S rRNA* (accession numbers provided in S1 Table). Sequences mined from BOLD and GenBank supplemented the molecular matrix from Bell et al. [37] in order to have all 425 families represented. Sequences for all four genes were available for a majority of families. Sequences were aligned for each gene separately using MUSCLE v.3.8.31 [38] and refined manually using MEGA v.6.05 [39]. The four gene alignments were then concatenated using SeqState v.1.4.1 [40].

Phylogenetic branch lengths were estimated on the Phylomatic cladogram from the molecular matrix by maximum likelihood using RAxML v.8.0.0 [29]. The RAxML output from the original 425 family maximum likelihood tree showed extreme molecular rate variation between 6 families (Apodanthaceae, Balanophoraceae, Corsiaceae, Hydnoraceae, Mitrastemonaceae and Rafflesiaceae) and their closest relatives. These large molecular differences can be explained by the fact that these are mostly parasitic lineages. Parasitism is an alternative evolutionary strategy in some plant families, and may cause the rapid erosion of sequences that may be highly conserved outside of these families [41]. For example, *rbcL* encodes the long-chain of the enzyme RuBisCO, a key component of carbon fixation, which is highly conserved in photosynthesizing plants, but has been lost or is highly divergent in parasitic plants [42]. Additionally, at least some of these families have been shown to exchange genetic information with their hosts, reducing our ability to infer their relatedness from DNA sequences [43]. Although Corsiaceae is not a parasitic family, but a myco-heterotroph, this alternate evolutionary strategy contributes similarly to the loss of photosynthetic ability and the erosion of genetic sequence information that is highly conserved in the rest of the seeded plants [44]. To reduce bias in branch length estimates, these families were removed and RAxML branch length estimation was executed on the reduced matrix.

Branch lengths were calibrated to millions of years using BEAST v.1.8.0 [30] and 26 fossil calibrations, enforced as minimum age constraints on the appropriate stem or crown nodes, following Bell et al. [37] and Smith et al. [45] (see S2 Table), keeping the branching topology fixed. First, we used PATHd8 [46] to generate a starting tree that satisfied the calibration constraints, and then branch lengths were estimated assuming log-normal priors with means of 5 million years and offset by the fossil date from S2 Table, and with the GTR+I+Γ site substitution model estimated for each of the four genes separately. This site substitution model was determined as the best fit to the data using ModelTest from the phangorn package in R [47]. The analysis in BEAST was run for 325 million generations, sampling every 50,000 generations. Sampling adequacy and model convergence was evaluated by examining parameter effective sample sizes (ESS values) and manually inspecting trace plots in Tracer [48]. We note that new discoveries will tend to push back fossil age estimates, for example, a new fossil age estimate for Nymphaeales [49] suggests the crown group for this clade could be several years older than the age used here; however, by enforcing calibrations as minimal age constraints and setting log-normal priors, our analyses allow for older estimates. A hard maximum age of 350 million years was enforced for the root of the phylogeny, reflecting the oldest reasonable divergence time between gymnosperms and angiosperms [50].

Last, the removed parasitic families were reinserted on the tree by introducing polytomies at the locations from which they were originally excised. These polytomies were then resolved using a BEAST input file generated with PolytomyResolver [51]. This script generates a pseudo-posterior distribution of phylogenies, by constraining the input tree topology and then resolving the introduced polytomies according to a birth-death model. The node heights of the final phylogeny are the median of this distribution, calculated in TreeAnnotator [30].

### Diversification Rate Calculations

Species richness estimates for 413 of the 425 seeded plant families were derived from the Plant List v.1.1 (http://www.theplantlist.org/), which reports the number of proposed species which have been accepted as unique species, rejected as synonyms, and have yet to be evaluated by relevant authorities for a given family. Family species richness was then estimated as:

$$S=S_{A}+\frac{S_{A}S_{U}}{S_{A}+S_{R}},$$

where S_A_ is the number of officially recognized species in a family, S_R_ is the number of species names that have been rejected as synonyms from that family and S_U_ is the number of unevaluated species from that family. In this way we were able to account for the fact that most plant families have not been completely evaluated in terms of species richness. For the remaining twelve families not included in the Plant List, published estimates of family species richness were obtained from the recent literature. Species richness estimates and sources are presented in S3 Table. The total species richness across all families (S_T_ = 367,831) conforms to current estimates of angiosperm global diversity [52].

We used information on stem age and species richness associated with each clade in the phylogeny to calculate two estimates of diversification rate (r), defined as the difference between the rate of speciation (λ) and the rate of extinction (μ) [8]. Following Magallón & Sanderson [8], we calculated two alternative estimates of diversification rate (r) according to the formula:

$$\hat{r}=\text{log}\left[S\left(1-\varepsilon \right)+\varepsilon \right]/t,$$

where ε is the relative extinction rate. The first assumed negligible extinction (ε = 0), and the second assumed a constant relative extinction rate (ε = μ/λ) of 0.9. At relative extinction rates greater than 0.9, it is suggested that speciation and extinction events would have to occur at a rate of more than 1 per million years [8], which is considered unlikely given published estimates of the frequency of these events. Therefore, these two estimates have been argued to represent reasonable upper and lower bounds on each clade’s net diversification rate [8].

We evaluated the time for speciation effect by fitting a linear model to the relationship between log (S) and clade age for adjacent 10 million year time windows, adjusting P-values for multiple tests using the Benjamini-Hochberg correction [53]. Clades over 100 million years in age were excluded from the analysis because the phylogeny becomes relatively node-poor and highly nested at this depth. To avoid the confounding effects of including clades nested within one another in the same subset of the data [4], clades with overlapping taxon sets were identified, and only one of which was included in a given analysis. This procedure was run twice, once removing the younger of the nested clades and then again removing the older of the nested clades. We chose this method rather than attempting to correct for phylogenetic non-independence, for example, by using a phylogenetic regression, because nested clades are by definition less species rich than the encompassing clades.

To evaluate the relationship between diversification rate and clade species richness, we modeled log(S) as a function of the net diversification rate (r) for all clades in the phylogeny.

Last, we identified major shifts in diversification rate across the tree using MEDUSA [54] in the geiger package in R [55], with the phylogeny and species richness estimates for each tip as inputs. MEDUSA first estimates a diversification rate model for the entire tree, and then adds a series of random break-points at which speciation and extinction rates are allowed to change. Alternative models are compared using AIC, with only the best performing break-point being retained. The process is repeated iteratively until the addition of new break-points no longer improves the sample-size corrected Akaike information criterion (AICc).

## Results

The 425 family phylogeny is available from the KNB Data Repository (doi:10.5063/F13T9F5P at https://knb.ecoinformatics.org/knb/d1/mn/v2/object/knb.1177.1). The timing of major divergence events align well with established molecular and fossil-based estimates, notably for angiosperms [37,56] and the entirety of the spermatophyte lineage [50].

**Fig 1:**
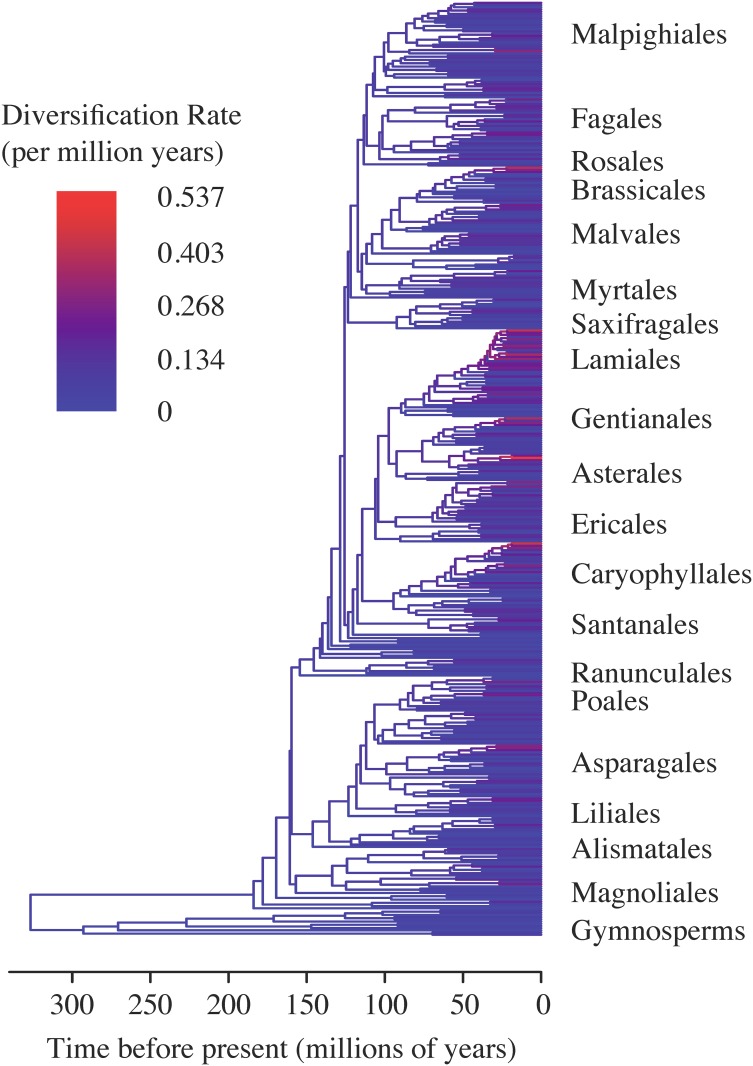
The reconstructed seeded plant phylogeny. Branches coloured according to the estimated diversification rate (assuming a relative extinction of ε = 0) of each clade following Magallón & Sanderson (2001). Rate estimates vary from 0/million years (blue) to 0.537/million years (red). Selected orders are labeled to aid interpretation.

Species richness, total diversification, and diversification rate estimation are reported for all 849 clades of the phylogeny (S4 Table). Absolute diversification (log(S)) ranged from 0 within single-species families to 12.82 for the entire phylogeny, with an average of 5.94. Estimates for net diversification rate varied greatly across the tree (Fig 1), and ranged from 0 to 0.537 per million years with a mean of 0.104 per million years when extinction was assumed to be negligible (ε = 0), and from 0 to 0.414 per million years with a mean of 0.067 per million years under our upper bound of relative extinction rate (ε = 0.9). We note that upper rate estimates should be interpreted cautiously, as they appear as obvious outliers (see also S1 Fig), a point we return to in the Discussion. Although the magnitude of extinction influenced the absolute estimates of diversification rate, changes to the rank order of clades were generally modest, with the rank of 8 of the top 10 fastest diversifying clades being conserved between the two models.

**Fig 2:**
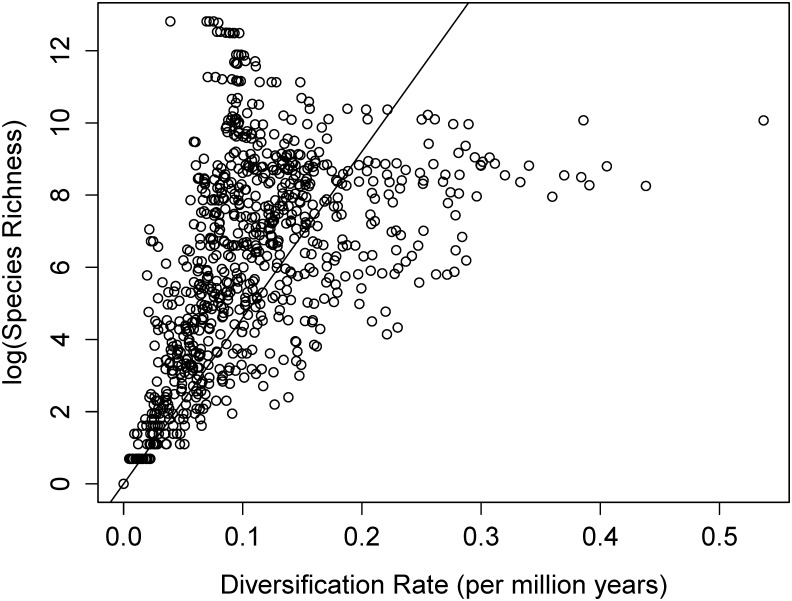
Logarithm of species richness plotted against net diversification rate (r) across all clades in the phylogeny. The correlation between log richness and diversification is highly significant (p<0.001, R^2^ = 0.77, from the linear regression).

Linear models of ln(S) and clade age within each of the 10 million year time windows are presented in Table 1. None of the models showed any significant relationship (p>0.10) between age and diversification rate after correcting for multiple tests. However, we find a highly significant relationship between log(S) and diversification rate (Fig 2, p<0.0001, R^2^ = 0.77).

**Table 1:** Linear models of clade age (million years: my) against ln(S) within 10 million year time windows.

Time window: subset younger node removed	Mean clade age (my)	Slope	Adjusted p	R^2^	Number of included nodes
20–30 my	26.9	0.218	0.477	0.044	48
30–40 my	36.1	-0.120	0.505	0.014	82
40–50 my	45.2	0.178	0.477	0.031	80
50–60 my	56.1	0.126	0.477	0.014	106
60–70 my	65.3	0.231	0.318	0.071	76
70–80 my	74.9	0.189	0.477	0.047	60
80–90 my	85.4	0.194	0.477	0.035	42
90–100 my	94.7	0.231	0.477	0.046	46
Time window: subset older node removed	Mean clade age (my)	Slope	Adjusted p	R^2^	Number of included nodes
20–30 my	25.2	0.081	0.689	0.009	62
30–40 my	35.0	-0.124	0.477	0.018	94
40–50 my	44.4	-0.041	0.773	0.000	89
50–60 my	55.4	-0.035	0.773	0.001	120
60–70 my	64.1	0.013	0.889	0.000	94
70–80 my	74.4	0.106	0.513	0.016	65
80–90 my	84.3	-0.054	0.773	0.003	52
90–100 my	94.3	0.100	0.717	0.008	51

**Fig 3:**
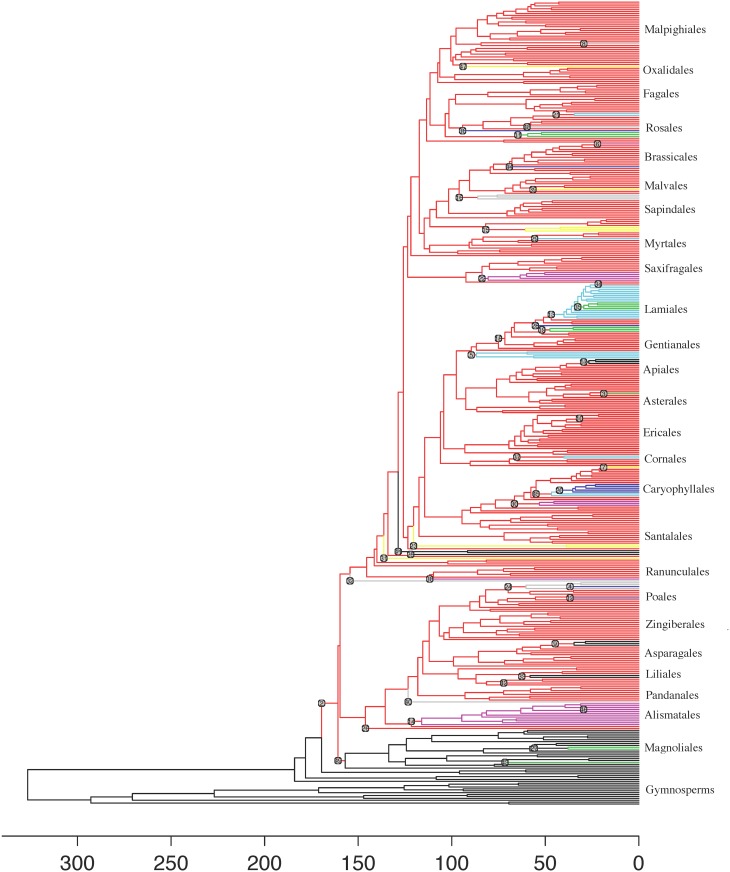
Results of the MEDUSA analysis of diversification rate-shifts on the reconstructed spermatophyte phylogeny. Numbered circles indicate the model number (see S5 Table) that corresponds with the model of diversification that was fit to the immediately descendent clade, with colour shading identifying the descendent lineages fitting to that model of diversification, allowing rate shifts within clades to be easily distinguished. Parameters associated with these models can be found in S5 Table. Horizontal scale bar represents time in millions of years.

The MEDUSA analysis assigned 49 different models to the phylogeny (Fig 3). Most of these models were of the Yule-process type, with only five of the fitted models including extinction >0 (S5 Table), although these five models cover a majority of the tree (Fig 3). Some of the models fit closely with specific taxonomic orders, including model 13 for Lamiales and model 14 for Alismatales.

## Discussion

We present here a new dated phylogenetic tree for spermatophytes that is completely sampled at the family level, and calibrated using molecular and fossil data with Bayesian inference. This phylogeny reflects expert consensus on plant phylogenetic relationships [28,32] along with robustly dated estimates of divergence times, and represents a valuable tool for comparative analysis. While several phylogenetic trees with greater density of sampling have been published recently (e.g. [31,57]), our reconstruction is notable in that it represents a complete sample of all seed plant families. Nonetheless, we note that the phylogenetic placement of Cynomoriaceae, Apodanthaceae and Vahliaceae remain controversial, and the evolutionary hypotheses presented here regarding these families should be used with caution. We used this new dated phylogeny of all seed plant families to contrast two alternative hypotheses for differences in species richness among higher taxa by exploring patterns of diversification across the spermatophyte family tree. Our results support previous analyses, illustrating large variation in diversification rates across spermatophyte lineages [7,15,58,59]. Monospecific families such as Barbeuiaceae, for example, have had similar length of time to diversify as species-rich groups such as Ericaceae.

Diversification rate estimates are sensitive to calibration of divergence times, number of recognized species within higher taxa, and phylogenetic accuracy. For example, the erroneous placement of a species-rich family towards the tips of the phylogeny will inflate diversification rate estimate for that family. This might occur if it is incorrectly placed as sister to an evolutionary distant family, or if branch lengths underestimate true divergence times because, for example, fossils are biased young or molecular rates have slowed. By constraining our analysis to a backbone phylogeny based upon a wealth of expert knowledge, we hope we have reduced impacts of phylogenetic error (but see caveats above). The pitfalls of relying upon a single or secondary calibration have been emphasized elsewhere (e.g. [60]). We attempted to provide robust estimates of divergence times by including multiple fossil calibrations, and taking median estimates from the Bayesian posterior distribution of ages, thus reducing sensitivity to outliers. It would also be possible to run all analyses across the posterior distribution of dated tree topologies, although we did not do this here. While we attempted to minimize error, we note that our higher net diversification rate estimates were greater than have been reported elsewhere for angiosperm taxa (e.g. [8,17]). However, these high rate estimates were associated with just a few lineages, and the vast majority of clades are characterized by rates falling well-with in the spectrum reported by Tank et al. [17] using similar methods on a differently sampled tree.

In our diversification rate analysis, we were interested in contrasting the time for speciation versus the ecological limits hypotheses of clade growth. We found no significant relationship between clade age and ln(S) in any of our 10 million year time windows, suggesting that time (stem clade age) is not an important predictor of species richness, at least for seeded plant clades older than twenty million years. Our results therefore do not support a simple time-for-speciation model, as also suggested by results from Salamin and Davies [61], Magallón and Castillo [15], and Tank et al. [17]. However, neither do we find support for a simplified version of Rabosky’s [9] model of ecological limits, in which we would expect the diversification of younger clades to be positively correlated to time, but older clades to show no relationship as they approach their ecological limits. It is, of course, possible that ecological limits might only be manifest when looking at more recent diversification; for which complete species-level phylogenies might be necessary. Unfortunately, we are still some way off from having a complete phylogeny for all c. 300,000 plant species; however, for species-rich animal clades, such as birds, for which complete phylogenies are now available [11], there is some intriguing evidence for density dependent diversification (e.g. [62,63]), that could be consistent with ecological limits.

Why did we not find strong evidence for either the time for speciation or ecological limits hypotheses? When we explore the fit of alternative diversification models to different clades across the phylogeny, we find that a few models fit to broad taxonomic groupings, such as the core eudicots, monocots and particular orders, but clades at lower levels of organization within these groups can follow models with radically different diversification rates. For example, the core eudicots are generally described by a model with a relatively low diversification rate (r = 0.050) and significant relative extinction (ε = 0.974), but the nested order Lamiales fits a model of much more rapid diversification (r = 0.228). As another example, the hyperdiverse monocot family Poaceae was fit to a model with a diversification rate (r = 0.256) more than eight times as fast as its closest relatives (Ecdeiocoleaceae, Joinvilleaceae and Flagellariaceae, r = 0.038). This pattern of nested radiations also matches to observations by Tank et al. [17], and likely helps explain why there is no strong support for either time or ecological limits in determining clade richness. However, we also note that a few simple models that cover a wide range of taxa can explain the species richness of most families. Much emphasis has been placed on rate variation across the plant tree of life (e.g. [7,8,15,16,17,37]); our results show that much of the spermatophyte family tree can be described by a few models, and are thus perhaps surprising.

The two models fit to the vast majority of the tree (models 1 and 2), have relative extinction values (ε = 0.993 and ε = 0.974, respectively) well above the reasonable upper limit proposed by Magallón and Sanderson [8] of ε = 0.9. A relative extinction rate this high would suggest an unfeasibly rapid turnover of lineages and imply a very large number of species extinctions for which there is no evidence in the fossil record. We suggest, therefore, that these results might indicate that assumptions of constant speciation and extinction rates may be violated. Although a model of non-constant rates could match to the asymptotic approach to an ecological limit, as suggested by Rabosky [9], it could equally result from fluctuating rates caused by changing ecological, environmental or geographic conditions through time [64]. Diversification rate variation might therefore reflect the contingent process of evolutionary diversification [65] whereby lineages with particular traits or key innovations are favoured in some environments or time periods. Thus it is possible that a lineage could originate but remain quite species poor until a changing ecological context allows its expansion sometime in the future. This may have occurred in mammals, for example [12,66]. Under this scenario, species richness is a product of the interaction between the life history traits of the clade and its ecological context [14].

There is a very large literature on seed plant diversification and, in particular the rise to ecological dominance of angiosperms (see recent review by Augusto et al. [67]). While the list of putative key traits associated with increased diversification is large, several have attracted particular attention, and can be grouped into two general classes: those that facilitate reproductive isolation and those that increase evolutionary rates. For example, modes of pollination and dispersal can enhance reproductive isolation through pollinator specificity or isolation by distance following long distance dispersal [68,69]. Growth form or generation time might directly influence evolutionary rates via cell generation times and rates of mitosis [70,71]. However, geographical extent is the best single predictor of clade species richness, with large-ranged clades characterized by higher diversification rates [72,73], although the direction of causation is unclear. In contrast, explanations for the low diversity of gymnosperms have focused on ε rather than *S*, and it is suggested that present day low richness reflects high extinction rather than low speciation rates [74]. Given this complexity, it may be unsurprising that we do not find strong support for any one hypothesis. Time for speciation is likely important, but clades possessing different key innovations may accumulate species at different rates. The link between clade richness and geographic extent provides some support for ecological limits, but traits, such as mode of dispersal, might also mediate this relationship.

While the MEDUSA analysis implemented here allows rate variation between clades, it fits birth-death models that assume underlying constant speciation and extinction rates within lineages, and Rabosky et al. [5] suggested that it is not appropriate to fit such models when there is no strong relationship between clade age and richness (but see Stadler et al. [75]). Alternative approaches allow more flexible models to be fit, for example, allowing time-dependent or diversity-dependent diversification processes (e.g. BAMM http://bamm-project.org/ [76] and RPANDA [77]), but their performance on higher-level phylogenies at the genus-level or family-level have not yet been well explored. It is possible to use a stochastic polytomy resolver, such as PASTIS [78], to return a set of completely resolved topologies given information on species richness of the terminal taxonomic units sampled in the tree. However, such approaches typically assume a constant birth-death process to resolve and, as a consequence, add bias to diversification rate analyses [25]. Because the MEDUSA algorithm is well suited for exploring diversification rates across higher taxa, it remains perhaps our best current option for describing the diversification of seed plants, but some underlying assumptions may be violated, and it is not yet clear whether rate estimates are thus unreliable. It will be interesting to explore additional methods that allow for more complex diversification models as more detailed phylogenetic trees become available.

Rabosky [9] and Wiens [4] have debated the validity of simple estimates of net diversification rate in explaining the variation in species richness across the tree of life. In Rabosky’s view, since we will observe most clades at their ecological limit of diversification, measures of net diversification rate are misleading because they will decrease over time [9]. Wiens [4] argues that as long as species richness is correlated to diversification rate, these metrics are still relevant to explaining patterns of species diversity. We show here that the two metrics are indeed correlated closely, indicating that, even if the underlying assumptions of these estimates are flawed, a significant proportion of the variation in species richness can be explained by net diversification rate. However, it is important to note that this relationship is to be expected because diversification rate is itself a function of clade species richness, and they are therefore not independent. When richness and clade age are randomized, the correlations are at least as good as the one observed from the data (mean R^2^ of 0.81 over 1000 runs). Consequently, estimates of net diversification rate will always capture some variation in species richness.

While we show diversification rate and species richness are inextricably intertwined, we argue that diversification rate remains an important metric for identifying contemporary clades that are undergoing rapid diversification versus clades that are species rich because they have had long to diversify. In addition, comparisons of relative rates allows us to separate species poor clades that have yet to diversify due to their young age from those which are species poor because they lack key innovations, ecological suitability and/or because of historical contingency.

## Supporting Information

S1 Fig Frequency histogram of clade net diversification rate estimates from Medusa. (PDF)

S2 Fig Complete seed plant phylogeny with internal nodes numbered. (PDF)

S1 Table Literature citations and/or Genbank accession numbers for DNA sequence data. (XLSX)

S2 Table Age constraints used to calibrate the phylogeny. (PDF)

S3 Table Species richness estimates for the 425 families in the phylogeny. (PDF)

S4 Table Diversification metrics and clade ages of all clades of the seeded plant phylogeny. (PDF)

S5 Table Model parameter estimates for the MEDUSA models of diversification. (PDF)

S6 Table List of clade members for each internal node of the seeded plant phylogeny. (CSV)

## References

[1] MacArthur RH. Patterns of species diversity. Biol Rev. 1965; 40(4): 510–533.

[2] Raup DM, Gould SJ, Schopf TJ, Simberloff DS. Stochastic models of phylogeny and the evolution of diversity. J Geol. 1973; 81(5): 525–542.

[3] Stanley SM. A theory of evolution above the species level. Proc Natl Acad Sci USA. 1975; 72(2): 646–650. 1054846 10.1073/pnas.72.2.646 PMC432371

[4] Wiens JJ. The causes of species richness patterns across space, time, and clades and the role of "ecological limits". Quart Rev Biol. 2011; 86(2): 75–96. 21800635 10.1086/659883

[5] Rabosky DL, Slater GJ, Alfaro ME. Clade age and species richness are decoupled across the eukaryotic tree of life. PLoS Biol. 2012; 10(8): e1001381 10.1371/journal.pbio.1001381 22969411 PMC3433737

[6] Slowinski JB, Guyer C. Testing whether certain traits have caused amplified diversification: An improved method based on a model of random speciation and extinction. Am Nat. 1993; 142(6): 1019–1024. 10.1086/285586 19425946

[7] Davies TJ, Barraclough TG, Chase MW, Soltis PS, Soltis DE, Savolainen V. Darwin's abominable mystery: Insights from a supertree of the angiosperms. Proc Natl Acad Sci USA. 2004; 101(7): 1904–1909. 10.1073/pnas.0308127100 14766971 PMC357025

[8] Magallón S, Sanderson MJ. Absolute diversification rates in angiosperm clades. Evolution, 2001; 55(9): 1762–1780. 11681732 10.1111/j.0014-3820.2001.tb00826.x

[9] Rabosky DL. Ecological limits and diversification rate: Alternative paradigms to explain the variation in species richness among clades and regions. Ecol Lett. 2009; 12(8): 735–743. 10.1111/j.1461-0248.2009.01333.x 19558515

[10] Pyron R, Wiens JJ. A large-scale phylogeny of Amphibia including over 2800 species, and a revised classification of extant frogs, salamanders, and caecilians. Mol Phyl Evol. 2011; 61: 543–583. 10.1016/j.ympev.2011.06.012 21723399

[11] Jetz W, Thomas GH, Joy JB, Hartmann K, Mooers AO. The global diversity of birds in space and time. Nature. 2012; 491(7424): 444–448. 10.1038/nature11631 23123857

[12] Stadler T, Rabosky DL, Ricklefs RE, Bokma F. On age and species richness of higher taxa. Am Nat. 2014; 184(4): 447–55. 10.1086/677676 25226180

[13] Bininda-Emonds OR, Cardillo M, Jones KE, MacPhee RD, Beck RM, Grenyer R, et al The delayed rise of present-day mammals. Nature. 2007; 446(7135): 507–512. 17392779 10.1038/nature05634

[14] Davies TJ, Barraclough TG, Savolainen V, Chase MW. Environmental causes for plant biodiversity gradients. Phil Trans R Soc B. 2004; 359(1450): 1645–1656. H2BJF1MJ3YAW29P1. 15519979 10.1098/rstb.2004.1524 PMC1693439

[15] Magallón S, Castillo A. Angiosperm diversification through time. Am J Bot. 2009; 96(1): 349–365. 10.3732/ajb.0800060 21628193

[16] Smith SA, Beaulieu JM, Stamatakis A, Donoghue MJ. Understanding angiosperm diversification using small and large phylogenetic trees. Am J Bot. 2011; 98(3): 404–14. 10.3732/ajb.1000481 21613134

[17] Tank DC, Eastman JM, Pennell MW, Soltis PS, Soltis DE, Hinchliff CE, et al Nested radiations and the pulse of angiosperm diversification: increased diversification rates often follow whole genome duplications. New Phytol. 2015; 207(2): 454–67. 10.1111/nph.13491 26053261

[18] Joly S, Davies TJ, Archambault A, Bruneau A, Derry A, Kembel SW, et al Ecology in the age of DNA barcoding: the resource, the promise and the challenges ahead. Mol Ecol Resources. 2014; 14(2): 221–32. 10.1111/1755-0998.12173 24118947

[19] de Queiroz A, Gatesy J. The supermatrix approach to systematics. Trends Ecol Evol. 2007; 22(1): 34–41. 17046100 10.1016/j.tree.2006.10.002

[20] Sanderson MJ, Purvis A, Henze C. Phylogenetic supertrees: assembling the trees of life. Trends Ecol Evol. 1998; 13(3): 105–109. 10.1016/S0169-5347(97)01242-1 21238221

[21] Bininda-Emonds OR ed. Phylogenetic supertrees: combining information to reveal the tree of life (Vol. 4). 2004; Springer Science & Business Media.

[22] Willis CG, Ruhfel B, Primack RB, Miller-Rushing AJ, Davis CC. Phylogenetic patterns of species loss in Thoreau's woods are driven by climate change. Proc Natl Acad Sci USA. 2008; 105(44): 17029–33. 10.1073/pnas.0806446105 18955707 PMC2573948

[23] Webb CO, Donoghue MJ. Phylomatic: Tree assembly for applied phylogenetics. Mol Ecol Notes. 2005 5(1): 181–183.

[24] Steel M, Mooers A. The expected length of pendant and interior edges of a Yule tree. Applied Math Lett. 2010; 23(11): 1315–1319.

[25] Rabosky DL. No substitute for real data: A cautionary note on the use of phylogenies from birth-death polytomy resolvers for downstream comparative analyses. Evolution. 2015; 69: 3207–3216. 10.1111/evo.12817 26552857

[26] Vos RA, Mooers AØ. Reconstructing divergence times for supertrees In Bininda-Emonds ORP editor Phylogenetic supertrees. Springer Netherlands 2004; pp. 281–299.

[27] Hinchliff CE, Smith SA, Allman JF, Burleigh JG, Chaudhary R, Coghill LM, et al Synthesis of phylogeny and taxonomy into a comprehensive tree of life. Proc Natl Acad Sci USA. 2015; 112:12764–12769. 10.1073/pnas.1423041112 26385966 PMC4611642

[28] Bremer B, Bremer K, Chase M, Fay M, Reveal J, Soltis D, et al An update of the angiosperm phylogeny group classification for the orders and families of flowering plants: APG III. Bot J Linn Soc. 2009; 161: 105–121.

[29] Stamatakis A. RAxML-VI-HPC: Maximum likelihood-based phylogenetic analyses with thousands of taxa and mixed models. Bioinformatics. 2006; 22(21): 2688–2690. 10.1093/bioinformatics/btl446 16928733

[30] Drummond AJ, Suchard MA, Xie D, Rambaut A. Bayesian phylogenetics with BEAUti and the BEAST 1.7. Mol Biol Evol. 2012; 29(8): 1969–1973. 10.1093/molbev/mss075 22367748 PMC3408070

[31] Zanne AE, Tank DC, Cornwell WK, Eastman JM, Smith SA, FitzJohn RG, et al Three keys to the radiation of angiosperms into freezing environments. Nature. 2014 506, 89–92, 10.1038/nature12872 24362564

[32] Stevens PF. Angiosperm Phylogeny Website. Version 12, 2001 onwards: http://www.mobot.org/MOBOT/research/APweb/.

[33] Byng JW, Chase MW, Christenhusz MJ, Fay MF, Judd WS, Mabberley DJ, et al An update of the Angiosperm Phylogeny Group classification for the orders and families of flowering plants: APG IV. Bot J Linn Soc. 2016; 181(1):1–20.

[34] Schaefer H, Renner SS. Phylogenetic relationships in the order Cucurbitales and a new classification of the gourd family (Cucurbitaceae). Taxon, 2011; 60(1): 122–138.

[35] Zhang Z, LI C, Li J. Phylogenetic placement of cynomorium in rosales inferred from sequences of the inverted repeat region of the chloroplast genome. J Syst Evol. 2009: 47(4): 297–304.

[36] Farjon A. World checklist and bibliography of conifers. 2001; Royal Botanical Gardens.

[37] Bell CD, Soltis DE, Soltis PS. The age and diversification of the angiosperms re-revisited. Am J Bot. 2010; 97(8): 1296–1303. 10.3732/ajb.0900346 21616882

[38] Edgar RC. MUSCLE: Multiple sequence alignment with high accuracy and high throughput. Nucleic Acids Res. 2004; 32(5): 1792–1797. 10.1093/nar/gkh340 15034147 PMC390337

[39] Kumar S, Nei M, Dudley J, Tamura K. MEGA: A biologist-centric software for evolutionary analysis of DNA and protein sequences. Briefings Bioinformatics. 2008; 9(4): 299–306. 10.1093/bib/bbn017 PMC2562624 18417537

[40] Müller K. Seqstate. Applied Bioinformatics. 2005; 4(1): 65–69. 16000015 10.2165/00822942-200504010-00008

[41] Bromham L, Cowman PF, Lanfear R. Parasitic plants have increased rates of molecular evolution across all three genomes. BMC Evol Biol. 2013; 13(1): 126 10.1186/1471-2148-13-126 23782527 PMC3694452

[42] Wolfe AD, de Pamphilis CW. The effect of relaxed functional constraints on the photosynthetic gene rbcL in photosynthetic and nonphotosynthetic parasitic plants. Mol Biol Evol. 1998; 15(10): 1243–1258. 9787431 10.1093/oxfordjournals.molbev.a025853

[43] Bock R. The give-and-take of DNA: Horizontal gene transfer in plants. Trends Plant Sci. 2010; 15(1): 11–22. 10.1016/j.tplants.2009.10.001 19910236

[44] Neinhuis C, Ibisch P. Corsiaceae In Kubitzki editor Flowering plants. monocotyledons. 1998; Springer, pp. 198–201.

[45] Smith SA, Beaulieu JM, Donoghue MJ. An uncorrelated relaxed-clock analysis suggests an earlier origin for flowering plants. Proc Natl Acad Sci. 2010; 107(13): 5897–902. 10.1073/pnas.1001225107 20304790 PMC2851901

[46] Britton T, Anderson C, Jaquet D, Lundqvist S, Bremer K. PATHd8-a new method for estimating divergence times in large phylogenetic trees without a molecular clock. 2006; Available from the Authors (www.math.su.se/PATHd8). 10.1080/10635150701613783 17886144

[47] Darriba D, Taboada GL, Doallo R, Posada D. ProtTest 3: fast selection of best-fit models of protein evolution. Bioinformatics. 2011; 27: 1164–1165. 10.1093/bioinformatics/btr088 21335321 PMC5215816

[48] Rambaut A, Drummond AJ (2007) Tracer v1.4, Available from http://beast.bio.ed.ac.uk/Tracer.

[49] Coiffard C, Mohr BA, Bernardes-de-Oliveira ME. *Jaguariba wiersemana* gen. nov. et sp. nov., an Early Cretaceous member of crown group Nymphaeales (Nymphaeaceae) from northern Gondwana. Taxon. 2013; 62(1): 141–51.

[50] Jiao Y, Wickett NJ, Ayyampalayam S, Chanderbali AS, Landherr L, Ralph P, et al Ancestral polyploidy in seed plants and angiosperms. Nature. 2011; 473(7345): 97–100. 10.1038/nature09916 21478875

[51] Kuhn TS, Mooers AØ, Thomas GH. A simple polytomy resolver for dated phylogenies. Methods Ecol Evol. 2011; 2(5): 427–436.

[52] Paton AJ, Brummitt N, Govaerts R, Harman K, Hinchcliffe S, Allkin B, et al Towards target 1 of the global strategy for plant conservation: A working list of all known plant species-progress and prospects. Taxon. 2008: 57(2): 602–611.

[53] Benjamini Y, Hochberg Y. Controlling the false discovery rate: a practical and powerful approach to multiple testing. J Roy Stat Soc. B. 1995; 57: 289–300.

[54] Alfaro ME, Santini F, Brock C, Alamillo H, Dornburg A, Rabosky DL et al Nine exceptional radiations plus high turnover explain species diversity in jawed vertebrates. Proc Natl Acad Sci USA. 2009; 106(32): 13410–13414. 10.1073/pnas.0811087106 19633192 PMC2715324

[55] R Core Team. R: A language and environment for statistical computing. R Foundation for Statistical Computing, Vienna, Austria 2015: http://www.R-project.org/.

[56] Crepet WL, Nixon KC, Gandolfo MA. Fossil evidence and phylogeny: The age of major angiosperm clades based on mesofossil and macrofossil evidence from cretaceous deposits. Am J Bot. 2004; 91(10): 1666–1682. 10.3732/ajb.91.10.1666 21652316

[57] Smith SA, Beaulieu JM, Donoghue MJ. Mega-phylogeny approach for comparative biology: an alternative to supertree and supermatrix approaches. BMC Evol Biol. 2009; 9: 37 10.1186/1471-2148-9-37 19210768 PMC2645364

[58] Fusco G, Cronk QC. A new method for evaluating the shape of large phylogenies. J Theor Biol. 1995: 175(2): 235–243.

[59] Silvestro D, Cascales-Miñana B, Bacon CD, Antonelli A. Revisiting the origin and diversification of vascular plants through a comprehensive Bayesian analysis of the fossil record. New Phyt. 2015; 207: 425–436. 10.1111/nph.13247 PMC4949670 25619401

[60] Graur D, Martin W. Reading the entrails of chickens: molecular timescales of evolution and the illusion of precision. TRENDS Genetics 2004; 20: 80–86. 10.1016/j.tig.2003.12.003 14746989

[61] Salamin N, Davies TJ. Using supertrees to investigate species richness in grasses and flowering plants In Bininda-Emonds ORP editor Phylogenetic supertrees. Springer Netherlands 2004 pp. 461–486.

[62] Phillimore AB, Price TD. Density-dependent cladogenesis in birds. PLoS Biol. 2008; 6: e71/. 10.1371/journal.pbio.0060071 18366256 PMC2270327

[63] Rabosky DL, Glor RE. Equilibrium speciation dynamics in a model adaptive radiation of island lizards. Proc Natl Acad Sci. 2010; 107(51): 22178–22183. 10.1073/pnas.1007606107 21135239 PMC3009809

[64] Davies TJ, Barraclough TG. The diversification of flowering plants through time and space: Key innovations, climate and chance In Hodkinson TR and Parnel JAN editors Reconstructing the tree of life: Taxonomy and systematics of species rich taxa. CRC Press 2007; pp. 149–160.

[65] de Queiroz A. Contingent predictability in evolution: key traits and diversification. Syst Biol. 2002; 51(6): 917–929. 12554458

[66] Kumar S, Hedges SB. A molecular timescale for vertebrate evolution. Nature. 1998; 392(6679): 917–920. 9582070 10.1038/31927

[67] Augusto L, Davies TJ, Delzon S, Schrijver A. The enigma of the rise of angiosperms: can we untie the knot?. Ecol Lett. 2014; 17(10):1326–1338. 10.1111/ele.12323 24975818

[68] Baker HG. Self-compatibility and establishment after 'long-distance' dispersal. Evolution. 1955; 9(3): 347–349.

[69] Eriksson O, Bremer B. Pollination systems, dispersal modes, life forms, and diversification rates in angiosperm families. Evolution. 1992; 46(1): 258–66. 28564968 10.1111/j.1558-5646.1992.tb02000.x

[70] Gaut BS, Morton BR, McCaig BC, Clegg MT. Substitution rate comparisons between grasses and palms: synonymous rate differences at the nuclear gene Adh parallel rate differences at the plastid gene rbcL. Proc Natl Acad Sci. 1996; 93(19): 10274–9. 8816790 10.1073/pnas.93.19.10274 PMC38374

[71] Lanfear R, Ho SY, Davies TJ, Moles AT, Aarssen L, Swenson NG, Warman L, et al Taller plants have lower rates of molecular evolution. Nature Com. 2013; 4: 1879. 10.1038/ncomms2836 23695673

[72] Davies TJ, Savolainen V, Chase MW, Goldblatt P, Barraclough TG. Environment, area, and diversification in the species?rich flowering plant family Iridaceae. Amer Nat. 2005; 166(3): 418–425. 16224695 10.1086/432022

[73] Vamosi JC, Vamosi SM. Key innovations within a geographical context in flowering plants: towards resolving Darwin's abominable mystery. Ecol Lett. 2010; 13: 1270–1279. 10.1111/j.1461-0248.2010.01521.x 20846343

[74] Leslie AB, Beaulieu JM, Rai HS, Crane PR, Donoghue MJ, Mathews S. Hemisphere-scale differences in conifer evolutionary dynamics. Proc Natl Acad Sci. 2012; 109(40): 16217–21. 10.1073/pnas.1213621109 22988083 PMC3479534

[75] Stadler T. Mammalian phylogeny reveals recent diversification rate shifts. Proc Natl Acad Sci USA. 2011; 108(15): 6187–6192. 10.1073/pnas.1016876108 21444816 PMC3076834

[76] Rabosky DL. Automatic detection of key innovations, rate shifts, and diversity-dependence on phylogenetic trees. PloS ONE. 2014; 9(2): p.e89543 10.1371/journal.pone.0089543 24586858 PMC3935878

[77] Morlon H, Condamine FL, Lewitus E, Manceau M. RPANDA: an R package for macroevolutionary analyses on phylogenetic trees. R package version 1.0. http://CRAN.R-project.org/package=RPANDA. 2015.

[78] Thomas GH, Hartmann K, Jetz W, Joy JB, Mimoto A, Mooers AO. PASTIS: an R package to facilitate phylogenetic assembly with soft taxonomic inferences. Methods Ecol Evol. 2013; 4(11): 1011–1017.

