# Correction: A Complete Fossil-Calibrated Phylogeny of Seed Plant Families as a Tool for Comparative Analyses: Testing the ‘Time for Speciation’ Hypothesis

**DOI:** 10.1371/journal.pone.0172816

**Published:** 2017-02-21

**Authors:** Liam W. Harris, T. Jonathan Davies

A reference is omitted from the first sentence of the last paragraph in the “Diversification Rate Calculations” section of the Methods. The sentence should read: “Last, we identified major shifts in diversification rate across the tree using MEDUSA [54] in the geiger (Pennell et al., 2014) package in R [55], with the phylogeny and species richness estimates for each tip as inputs.”

The reference is: Pennell MW, Eastman JM, Slater GJ, Brown JW, Uyeda JC, FitzJohn RG, Alfaro ME, Harmon LJ. geiger v2. 0: an expanded suite of methods for fitting macroevolutionary models to phylogenetic trees. Bioinformatics. 2014 10:btu181.

The number of independent calibration points in the phylogenetic dating, as detailed in S2 Table, appears incorrectly throughout the article as 26 calibrations. The correct number of fossil calibrations is 29.

The prior type appears incorrectly in the fifth paragraph in the “Phylogeny Reconstruction” section of the Methods. “Log-normal” should be listed as “exponential”.
