# Supplementary material for: A Complete Fossil-Calibrated Phylogeny of Seed Plant Families as a Tool for Comparative Analyses: Testing the ‘Time for Speciation’ Hypothesis
Source: PLoS One. 2016 Oct 5;11(10):e0162907. doi: 10.1371/journal.pone.0162907 (PMC5051821; doi:10.1371/journal.pone.0162907)

Figure S1. Frequency histogram of clade net diversification rate estimates from Medusa (data from Table S4). Grey bars with models fitted assuming a relative extinction rate (ε) of 0.9, and pink bars with ε = 0.

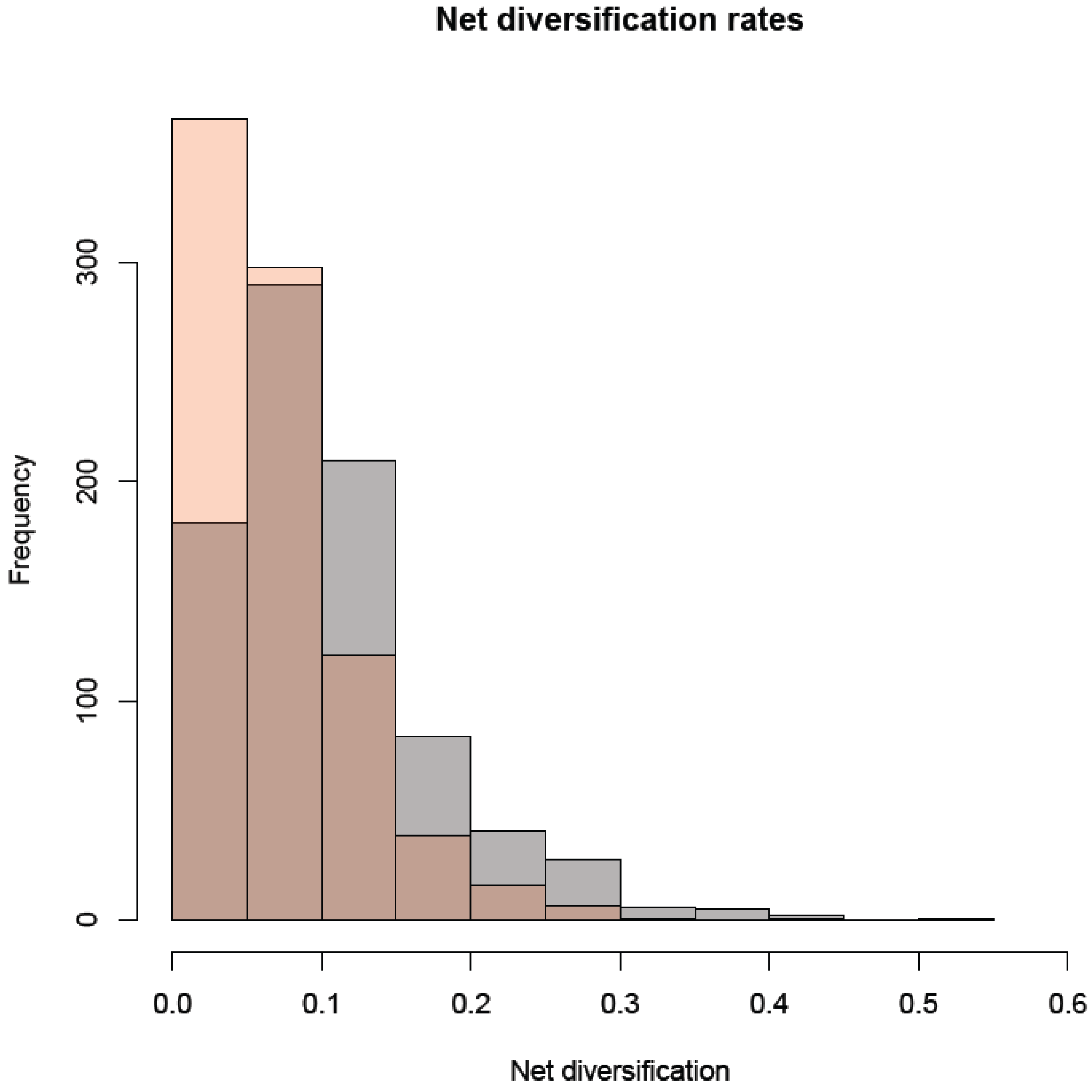

Supplement: S1 Fig — (PDF) [file pone.0162907.s001.pdf]
