# Supplementary figures and images for: A Complete Fossil-Calibrated Phylogeny of Seed Plant Families as a Tool for Comparative Analyses: Testing the ‘Time for Speciation’ Hypothesis
Source: PLoS One. 2016 Oct 5;11(10):e0162907. doi: 10.1371/journal.pone.0162907 (PMC5051821; doi:10.1371/journal.pone.0162907)

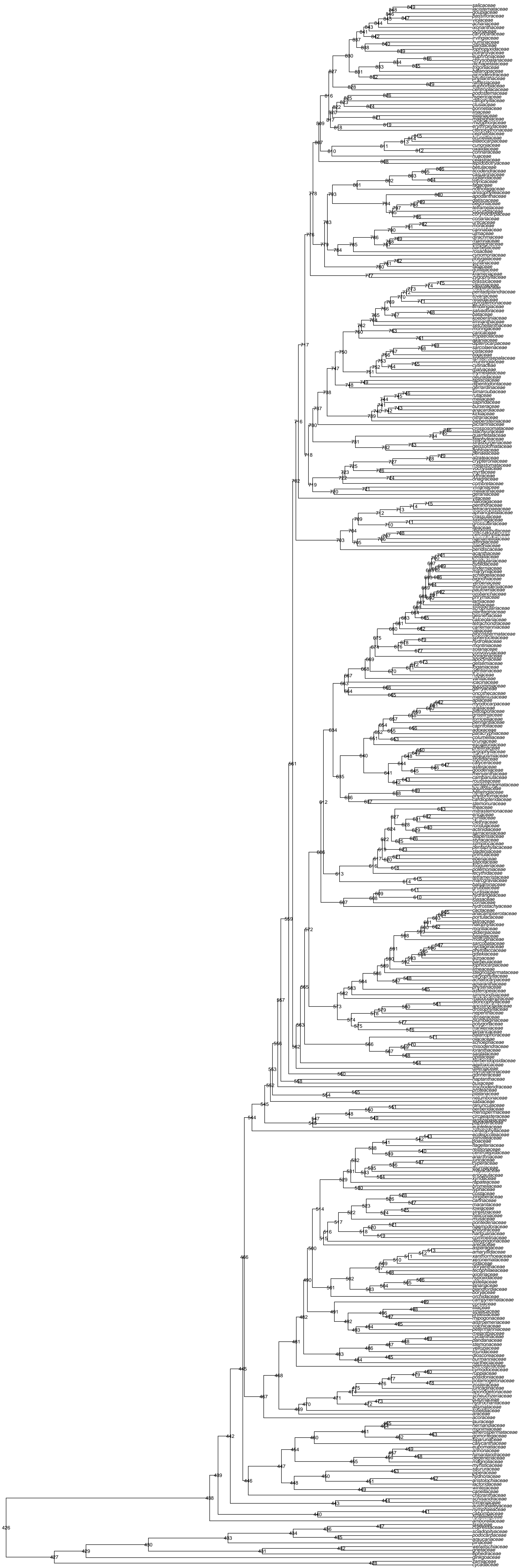

300

250

200

150

100

50

0

Supplement: S2 Fig — (PDF) [file pone.0162907.s002.pdf]
