# Supplementary material for: A Complete Fossil-Calibrated Phylogeny of Seed Plant Families as a Tool for Comparative Analyses: Testing the ‘Time for Speciation’ Hypothesis
Source: PLoS One. 2016 Oct 5;11(10):e0162907. doi: 10.1371/journal.pone.0162907 (PMC5051821; doi:10.1371/journal.pone.0162907)
Supplement: S2 Table — (PDF) [file pone.0162907.s004.pdf]

Table S2: Age constraints used to calibrate the phylogeny to time in PATHd8 and BEAST

Clade	Minimum Age	Source	Crown or Stem
Angiosperms	132	Bell et al. 2010	Crown
Aquifoliales	85	Smith et al. 2010	Crown
Burseraceae/Anacardiaceae	50	Bell et al. 2010	Crown
Buxales	112	Smith et al. 2010	Stem
Canellales	122.5	Smith et al. 2010	Stem
Caryophyllales	83.5	Bell et al. 2010	Crown
Clusia/Hypericum	89	Bell et al. 2010	Crown
Cornales	86	Bell et al. 2010	Crown
Ericales	91.2	Bell et al. 2010	Crown
Fabales	59.9	Bell et al. 2010	Crown
Fagales	96	Smith et al. 2010	Stem
Gunnerales	88.2	Smith et al. 2010	Crown
Gymnosperms	290	Smith et al. 2010	Crown
Lamiales	44.3	Bell et al. 2010	Crown
Laurales	108.8	Bell et al. 2010	Crown
Magnoliales	108.8	Smith et al. 2010	Stem
malpighiales	49	Bell et al. 2010	Crown
Malvales	65.5	Bell et al. 2010	Crown
Myrtales	88.2	Bell et al. 2010	Crown
Nymphaeales	105	Smith et al. 2010	Crown
Pandanales	65	Bell et al. 2010	Crown
Poales	68.1	Bell et al. 2010	Crown
Proteales	98	Bell et al. 2010	Crown
Santalales	51.9	Bell et al. 2010	Crown
Sapidales	65	Bell et al. 2010	Crown
Saxifragales	89.3	Smith et al. 2010	Crown
Simaroubaceae/Rutaceae	50	Bell et al. 2010	Crown
Solanales	44.3	Bell et al. 2010	Crown
Zingiberales	83.5	Bell et al. 2010	Crown

Bell CD, Soltis DE, Soltis PS. The age and diversification of the angiosperms re-revisited. Am J Bot. 2010; 97(8): 1296-1303. doi:10.3732/ajb.0900346.

Smith SA, Beaulieu JM, Donoghue MJ. An uncorrelated relaxed-clock analysis suggests an earlier origin for flowering plants. Proc Natl Acad Sci. 2010; 107(13): 5897-902.
