# Supplementary material for: A Complete Fossil-Calibrated Phylogeny of Seed Plant Families as a Tool for Comparative Analyses: Testing the ‘Time for Speciation’ Hypothesis
Source: PLoS One. 2016 Oct 5;11(10):e0162907. doi: 10.1371/journal.pone.0162907 (PMC5051821; doi:10.1371/journal.pone.0162907)
Supplement: S3 Table — (PDF) [file pone.0162907.s005.pdf]

Table S3: Species richness estimates for the 425 families in the phylogeny, calculated using the presented data mined from the Plant List, unless otherwise indicated.

Family	Accepted	Rejected	Unclassified	Plant List Estimate	Alternative Estimate	Alternative Source
Acanthaceae	3947	3660	5162	6625		
Achariaceae	101	115	218	203		
Achatocarpaceae	11	6	0	11		
Acoraceae	2	34	3	2		
Actinidiaceae	176	177	277	314		
Adoxaceae	194	179	280	340		
Aextoxicaceae	1	0	0	1		
Aizoaceae	2271	1327	2083	3586		
Akaniaceae	2	5	0	2		
Alismataceae	120	359	23	126		
Alseuosmiaceae	6	8	11	11		
Alstroemeriaceae	259	365	33	273		
Altingiaceae	19	17	4	21		
Alzateaceae	NA	NA	NA	NA	1	Schönenberger & Conti (2003)
Amaranthaceae	2052	3450	2369	2936		
Amaryllidaceae	2258	4133	374	2390		
Amborellaceae	1	0	0	1		
Anacampserotaceae	58	46	32	76		
Anacardiaceae	701	754	1479	1414		
Anarthriaceae	11	10	1	12		
Ancistrocladaceae	21	14	1	22		
Anisophylleaceae	39	16	9	45		
Annonaceae	2106	1779	1236	2776		
Aphanopetalaceae	2	2	0	2		
Aphloiaceae	2	16	4	2		
Apiaceae	3257	5070	4837	5149		
Apocynaceae	5556	8848	3841	7038		
Apodanthaceae	26	13	6	30		
Aponogetonaceae	58	39	3	60		
Aquifoliaceae	480	252	407	747		
Araceae	3368	3392	1345	4038		
Araliaceae	1533	2344	435	1705		
Araucariaceae	39	117	7	41		
Arecaceae	2522	4443	376	2658		
Argophyllaceae	22	4	3	25		
Aristolochiaceae	624	542	207	735		
Asparagaceae	2929	6392	584	3113		

Asteliaceae	37	56	0	37		
Asteraceae	NA	NA	NA	NA	23600	APG III (2009)
Asteropeiaceae	8	3	0	8		
Atherospermataceae	20	16	7	24		
Austrobaileyaceae	1	1	0	1		
Balanopaceae	9	8	0	9		
Balanophoraceae	45	173	27	51		
Balsaminaceae	488	154	742	1052		
Barbeuiaceae	1	0	0	1		
Barbeyaceae	1	0	0	1		
Basellaceae	19	66	3	20		
Bataceae	2	2	3	4		
Begoniaceae	1601	967	219	1738		
Berberidaceae	755	426	336	970		
Berberidopsidaceae	2	1	1	3		
Betulaceae	234	622	108	264		
Biebersteiniaceae	5	6		5		
Bignoniaceae	852	2453	322	935		
Bixaceae	23	67	6	25		
Blandfordiaceae	4	9	1	4		
Bonnetiaceae	36	20	21	50		
Boraginaceae	2686	3858	3993	4325		
Boryaceae	12	11	2	13		
Brassicaceae	4060	8948	2693	4901		
Bromeliaceae	3320	3194	300	3473		
Brunelliaceae	60	15	7	66		
Bruniaceae	92	152	71	119		
Burmanniaceae	163	193	3	164		
Burseraceae	649	885	367	804		
Butomaceae	2	4	0	2		
Buxaceae	122	90	36	143		
Byblidaceae	7	3	0	7		
Cabombaceae	6	18	2	7		
Cactaceae	2233	5422	5504	3839		
Calceolariaceae	281	458	55	302		
Calophyllaceae	127	69	184	246		
Calycanthaceae	10	50	4	11		
Calyceraceae	56	86	19	63		
Campanulaceae	2385	4492	304	2490		
Campynemataceae	4	2	0	4		
Canellaceae	21	14	3	23		
Cannabaceae	102	383	151	134		
Cannaceae	12	164	9	13		
Capparaceae	449	731	306	565		

Caprifoliaceae	857	1272	1947	1641		
Cardiopteridaceae	46	88	24	54		
Caricaceae	47	126	17	52		
Carlemanniaceae	5	3	1	6		
Caryocaraceae	26	31	2	27		
Caryophyllaceae	2456	3928	4247	4090		
Casuarinaceae	91	102	5	93		
Celastraceae	1168	1431	1339	1770		
Centrolepidaceae	36	62	2	37		
Centroplacaceae	8	5	4	10		
Cephalotaceae	1	0	0	1		
Ceratophyllaceae	4	42	1	4		
Cercidiphyllaceae	2	1	1	3		
Chloranthaceae	71	85	28	84		
Chrysobalanaceae	535	563	23	546		
Circaeasteraceae	2	0	0	2		
Cistaceae	201	435	671	413		
Cleomaceae	257	336	171	331		
Clethraceae	85	134	17	92		
Clusiaceae	1047	539	385	1301		
Colchicaceae	284	542	18	290		
Columelliaceae	8	18	0	8		
Combretaceae	480	693	613	731		
Commelinaceae	728	1368	83	757		
Connaraceae	267	500	254	355		
Convolvulaceae	1296	1596	3121	2695		
Coriariaceae	16	16	0	16		
Cornaceae	124	523	37	131		
Corsiaceae	27	1	0	27		
Corynocarpaceae	5	2	0	5		
Costaceae	139	168	7	142		
Crassulaceae	1482	2329	1746	2161		
Crossosomataceae	8	13	2	9		
Crypteroniaceae	NA	NA	NA	NA	10	Conti et al. 2002
Ctenolophonaceae	2	2	0	2		
Cucurbitaceae	965	1680	1074	1357		
Cunoniaceae	245	167	345	450		
Cupressaceae	166	991	46	173		
Curtisiaceae	1	6	0	1		
Cycadaceae	92	70	7	96		
Cyclanthaceae	231	140	31	250		
Cymodoceaceae	16	64	2	16		
Cynomoriaceae	1	2	0	1		
Cyperaceae	5784	12391	637	5987		

Cyrillaceae	8	20	4	9		
Cytinaceae	11	24	3	12		
Daphniphyllaceae	34	61	2	35		
Dasypogonaceae	16	3	1	17		
Datiscaceae	2	6	1	2		
Degeneriaceae	2	0	0	2		
Diapensiaceae	12	22	21	19		
Dichapetalaceae	196	286	47	215		
Didiereaceae	22	16	2	23		
Dilleniaceae	219	250	579	489		
Dioncophyllaceae	3	0	2	5		
Dioscoreaceae	653	721	55	679		
Dipentodontaceae	21	18	4	23		
Dipterocarpaceae	147	234	843	472		
Dirachmaceae	2	0	0	2		
Doryanthaceae	2	3	0	2		
Droseraceae	189	188	10	194		
Drosophyllaceae	1	3	1	1		
Ebenaceae	751	876	71	784		
Ecdeiocoleaceae	3	0	0	3		
Elaeagnaceae	108	100	42	130		
Elaeocarpaceae	644	406	297	826		
Elatinaceae	57	85	32	70		
Emblingiaceae	1	0	0	1		
Ephedraceae	70	92	2	71		
Ericaceae	3554	4646	3889	5240		
Eriocaulaceae	1206	1074	31	1222		
Erythroxylaceae	267	146	36	290		
Escallioniaceae	55	111	118	94		
Eucommiaceae	1	0	0	1		
Euphorbiaceae	6547	9874	489	6742		
Euphroniaceae	4	3	1	5		
Eupomatiaceae	3	0	0	3		
Eupteleaceae	2	4	1	2		
Fabaceae	NA	NA	NA	NA	19400	Judd et al. 2002
Fagaceae	1101	2824	216	1162		
Flagellariaceae	4	5	3	5		
Fouquieriaceae	12	8	4	14		
Frankeniaceae	73	88	16	80		
Garryaceae	25	23	4	27		
Geissolomataceae	1	1	1	2		
Gelsemiaceae	13	42	16	17		
Gentianaceae	1682	2923	1357	2178		
Geraniaceae	841	912	2114	1855		

Gerrardinaceae	2	0	0	2		
Gesneriaceae	3122	2924	1844	4074		
Ginkgoaceae	1	6	1	1		
Gisekiaceae	7	15	2	8		
Gnetaceae	41	67	10	45		
Gomortegaceae	1	3	0	1		
Goodeniaceae	329	194	354	552		
Goupiaceae	2	4	1	2		
Griseliniaceae	NA	NA	NA	NA	7	APG III (2009)
Grossulariaceae	195	153	344	388		
Grubbiaceae	3	8	5	4		
Guamatelaceae	NA	NA	NA	NA	1	APG III (2009)
Gunneraceae	69	37	5	72		
Gyrostemonaceae	17	23	13	23		
Haemodoraceae	101	94	6	104		
Halophytaceae	1	1	0	1		
Haloragaceae	92	111	169	169		
Hamamelidaceae	99	73	63	135		
Hanguanaceae	10	8	0	10		
Haptanthaceae	1	0	0	1		
Heliconiaceae	204	176	9	209		
Helwingiaceae	4	8	0	4		
Hernandiaceae	51	40	40	73		
Himantandraceae	1	8	0	1		
Huaceae	4	0	2	6		
Humiriaceae	63	68	24	75		
Hydatellaceae	12	7	0	12		
Hydnoraceae	10	21	1	10		
Hydrangeaceae	237	224	193	336		
Hydrocharitaceae	133	414	23	139		
Hydroleaceae	11	41	33	18		
Hydrostachyaceae	19	10	8	24		
Hypericaceae	584	772	359	739		
Hypoxidaceae	154	292	25	163		
Icacinaceae	212	247	89	253		
Iridaceae	2315	4512	319	2423		
Irvingiaceae	11	60	2	11		
Iteaceae	19	17	16	27		
Ixioliriaceae	4	17	0	4		
Ixonanthaceae	20	10	39	46		
Joinvilleaceae	4	5	0	4		
Juglandaceae	89	142	169	154		
Juncaceae	506	847	58	528		
Juncaginaceae	35	74	16	40		

Kirkiaceae	5	2	5	9		
Koeberliniaceae	2	0	0	2		
Krameriaceae	25	27	3	26		
Lacistemataceae	13	35	13	17		
Lactoridaceae	1	0	0	1		
Lamiaceae	7886	13178	1490	8444		
Lanariaceae	1	7	0	1		
Lardizabalaceae	37	33	15	45		
Lauraceae	2978	2512	2035	4082		
Lecythidaceae	341	808	37	352		
Lentibulariaceae	312	448	195	392		
Lepidobotryaceae	2	0	0	2		
Liliaceae	746	1295	88	778		
Limeaceae	20	15	39	42		
Limnanthaceae	9	12	8	12		
Linaceae	213	199	345	391		
Linderniaceae	164	259	425	329		
Loasaceae	314	320	165	396		
Loganiaceae	351	371	470	579		
Lophiocarpaceae	5	16	4	6		
Lophopyxidaceae	NA	NA	NA	NA	2	Watson & Dallwitz 2013
Loranthaceae	886	1741	1526	1401		
Lowiaceae	17	6	1	18		
Lythraceae	604	685	533	854		
Magnoliaceae	250	672	78	271		
Malpighiaceae	1301	1129	598	1621		
Malvaceae	4465	5190	4856	6711		
Marantaceae	569	907	77	599		
Marcgraviaceae	137	105	48	164		
Martyniaceae	21	29	24	31		
Mayacaceae	6	16	2	7		
Melanthiaceae	181	442	16	186		
Melastomataceae	4079	2477	5847	7717		
Meliaceae	669	1861	668	846		
Melianthaceae	20	66	4	21		
Menispermaceae	448	585	636	724		
Menyanthaceae	55	73	117	105		
Metteniusaceae	3	2	4	5		
Misodendraceae	8	13	21	16		
Mitrastemonaceae	2	4	2	3		
Molluginaceae	103	82	111	165		
Monimiaceae	134	89	345	341		
Montiaceae	113	249	106	146		
Montiniaceae	4	1	1	5		

Moraceae	1217	2880	778	1448		
Moringaceae	13	5	32	36		
Muntingiaceae	3	1	1	4		
Musaceae	78	152	5	80		
Myodocarpaceae	15	13	0	15		
Myricaceae	53	70	92	93		
Myristicaceae	170	206	768	517		
Myrothamnaceae	2	1	0	2		
Myrtaceae	5970	7475	544	6212		
Nartheciaceae	36	67	1	36		
Nelumbonaceae	2	15	28	5		
Nepenthaceae	7	5	247	151		
Neuradaceae	7	9	3	8		
Nitrariaceae	12	15	11	17		
Nothofagaceae	38	63	7	41		
Nyctaginaceae	450	540	430	645		
Nymphaeaceae	70	145	252	152		
Ochnaceae	560	577	961	1033		
Olacaceae	149	181	168	225		
Oleaceae	688	1867	106	717		
Onagraceae	832	1504	954	1172		
Oncothecaceae	1	0	3	4		
Opiliaceae	33	78	16	38		
Orchidaceae	27801	39992	2595	28865		
Orobanchaceae	1613	1443	2344	2850		
Oxalidaceae	601	931	688	871		
Paeoniaceae	36	128	15	39		
Pandaceae	17	51	8	19		
Pandanaceae	1062	627	21	1075		
Papaveraceae	920	1099	699	1239		
Paracryphiaceae	29	12	9	35		
Passifloraceae	932	790	527	1217		
Paulowniaceae	20	21	14	27		
Pedaliaceae	67	43	84	118		
Penaeaceae	45	51	96	90		
Pennantiaceae	NA	NA	NA	NA	2	Mabberley 2008
Pentadiplandraceae	1	3	1	1		
Pentaphragmataceae	2	5	38	13		
Pentaphylacaceae	429	389	269	570		
Penthoraceae	2	2	1	3		
Peridiscaceae	9	2	2	11		
Petermanniaceae	1	0	0	1		
Petrosaviaceae	4	8	0	4		
Phellinaceae	NA	NA	NA	NA	12	APG III (2009)

Philesiaceae	2	6	1	2
Philydraceae	6	8	1	6
Phrymaceae	199	96	211	341
Phyllanthaceae	2099	3150	179	2171
Phyllonomaceae	5	6	0	5
Physenaceae	2	0	0	2
Phytolaccaceae	62	149	87	88
Picramniaceae	48	42	25	61
Picrodendraceae	96	105	10	101
Pinaceae	255	1209	66	266
Piperaceae	2658	2121	2344	3962
Pittosporaceae	170	147	302	332
Plantaginaceae	1614	1989	3450	3159
Platanaceae	9	38	3	10
Plocospermataceae	1	4	1	1
Plumbaginaceae	635	630	1075	1175
Poaceae	11554	32806	3065	12352
Podocarpaceae	191	432	27	199
Podostemaceae	250	197	273	403
Polemoniaceae	455	428	631	780
Polygalaceae	1163	584	1123	1911
Polygonaceae	1384	1880	2120	2283
Pontederiaceae	33	167	6	34
Portulacaceae	258	384	243	356
Posidoniaceae	10	11	6	13
Potamogetonaceae	186	497	53	200
Primulaceae	2788	3140	2195	3820
Proteaceae	1252	866	2238	2575
Putranjivaceae	216	229	8	220
Quillajaceae	2	2	9	7
Rafflesiaceae	22	10	22	37
Ranunculaceae	2377	3260	3422	3820
Rapateaceae	95	34	3	97
Resedaceae	51	110	162	102
Restionaceae	482	676	12	487
Rhabdodendraceae	3	12	0	3
Rhamnaceae	839	827	1252	1470
Rhipogonaceae	6	5	0	6
Rhizophoraceae	142	180	97	185
Roridulaceae	2	4	0	2
Rosaceae	4828	7041	16996	11742
Rousseaceae	14	8	1	15
Rubiaceae	13673	16393	1288	14259
Ruppiaceae	8	28	0	8

Rutaceae	1730	2634	2318	2649
Sabiaceae	116	98	158	202
Salicaceae	1269	1711	2141	2181
Salvadoraceae	8	17	33	19
Santalaceae	992	743	699	1392
Sapindaceae	1751	1559	1571	2582
Sapotaceae	1343	3401	176	1393
Sarcobataceae	2	2	2	3
Sarcolaenaceae	69	28	5	73
Sarraceniaceae	32	8	64	83
Saururaceae	7	18	2	8
Saxifragaceae	775	924	1073	1264
Scheuchzeriaceae	1	5	0	1
Schisandraceae	73	83	25	85
Schlegeliaceae	43	24	6	47
Schoepfiaceae	51	47	31	67
Sciadopityaceae	1	3	0	1
Scrophulariaceae	1576	1017	2396	3032
Setchellanthaceae	1	0	0	1
Simaroubaceae	121	152	127	177
Simmondsiaceae	2	0	3	5
Siparunaceae	61	198	8	63
Sladeniaceae	3	0	0	3
Smilacaceae	261	431	40	276
Solanaceae	2678	2592	3128	4268
Sphaerosepalaceae	20	6	3	22
Sphenocleaceae	2	8	1	2
Stachyuraceae	7	8	8	11
Staphyleaceae	30	48	56	52
Stegnospermataceae	5	1	0	5
Stemonaceae	36	33	5	39
Stemonuraceae	71	183	8	73
Stilbaceae	38	103	10	41
Strasburgeriaceae	2	1	1	3
Strelitziaceae	7	31	1	7
Stylidiaceae	31	109	348	108
Styracaceae	133	184	118	183
Surianaceae	6	6	2	7
Symplocaceae	210	341	552	420
Talinaceae	28	56	34	39
Tamaricaceae	79	78	134	146
Tapisciaceae	6	1	0	6
Taxaceae	31	126	4	32
Tecophilaeaceae	26	48	3	27

Tetracarpaeaceae	NA	NA	NA	NA	1	Kubitzki 2007
Tetrachondraceae	3	5	2	4		
Tetramelaceae	1	2	5	3		
Tetrameristaceae	2	0	4	6		
Theaceae	370	468	264	487		
Thomandersiaceae	NA	NA	NA	NA	6	Wortley et al. 2007
Thurniaceae	4	7	0	4		
Thymelaeaceae	938	1121	551	1189		
Ticodendraceae	1	0	0	1		
Tofieldiaceae	29	110	0	29		
Torricelliaceae	8	9	2	9		
Tovariaceae	2	1	0	2		
Trigoniaceae	32	20	17	42		
Trimeniaceae	NA	NA	NA	NA	5	Watson & Dallwitz 2013
Triuridaceae	54	86	1	54		
Trochodendraceae	2	1	0	2		
Tropaeolaceae	89	117	78	123		
Typhaceae	65	143	10	68		
Ulmaceae	64	109	134	114		
Urticaceae	1465	1491	1961	2437		
Vahliaceae	5	24	9	7		
Velloziaceae	278	227	42	301		
Verbenaceae	1035	1495	182	1109		
Violaceae	806	692	1811	1780		
Vitaceae	985	1255	785	1330		
Vivianiaceae	16	33	41	29		
Vochysiaceae	215	106	77	267		
Welwitschiaceae	1	2	0	1		
Winteraceae	32	78	131	70		
Xanthorrhoeaceae	1236	1854	254	1338		
Xeronemataceae	2	1	0	2		
Xyridaceae	387	228	17	398		
Zamiaceae	216	241	21	226		
Zingiberaceae	1587	1658	101	1636		
Zosteraceae	23	30	2	24		
Zygophyllaceae	211	207	345	385		
