# Supplementary material for: A Complete Fossil-Calibrated Phylogeny of Seed Plant Families as a Tool for Comparative Analyses: Testing the ‘Time for Speciation’ Hypothesis
Source: PLoS One. 2016 Oct 5;11(10):e0162907. doi: 10.1371/journal.pone.0162907 (PMC5051821; doi:10.1371/journal.pone.0162907)
Supplement: S4 Table — (PDF) [file pone.0162907.s006.pdf]

Table S4: Diversification metrics and clade ages of all clades of the seeded plant phylogeny. The first 425 clade numbers refers to tips of the phylogeny as they are listed in Fig S2 from bottom to top. The remainder refer to internal nodes labeled in Fig S2. Families descended from each node are listed in S6 Table.

Clade Number	Species Richness	Diversification (Ω)	Clade age	Diversification rate (ε=0)	Diversification rate (ε=0.9)
1	96	4.564348191	69.46613322	0.065706093	0.033849232
2	226	5.420534999	69.46613322	0.078031333	0.045446612
3	1	0	270.6661112	0	0
4	71	4.262679877	147.0862728	0.028980814	0.014137564
5	45	3.80666249	91.75330086	0.041488017	0.018379709
6	1	0	91.75330086	0	0
7	266	5.583496309	171.1536964	0.032622704	0.0193638
8	41	3.713572067	93.84741488	0.039570318	0.017149518
9	199	5.293304825	93.84741488	0.05640331	0.032339228
10	1	0	101.4737174	0	0
11	173	5.153291594	64.39035264	0.080032045	0.04505988
12	32	3.465735903	64.39035264	0.053823838	0.021913018
13	1	0	183.9906694	0	0
14	12	2.48490665	108.2887884	0.022947035	0.00685147
15	7	1.945910149	32.54101524	0.059798692	0.014443422
16	152	5.023880521	32.54101524	0.154386103	0.085394363
17	1	0	95.85730438	0	0
18	5	1.609437912	60.43091222	0.026632693	0.005567883
19	85	4.442651256	60.43091222	0.073516204	0.037078866
20	84	4.430816799	156.9385143	0.02823282	0.014209478
21	23	3.135494216	77.26194415	0.040582647	0.015054641
22	70	4.248495242	77.26194415	0.0549882	0.026751369
23	1	0	71.56365766	0	0
24	735	6.599870499	26.70065453	0.247180102	0.161398888
25	10	2.302585093	26.70065453	0.086237028	0.024038882
26	3962	8.284504227	54.26945809	0.152655002	0.110268065
27	8	2.079441542	54.26945809	0.038316976	0.009777659
28	517	6.248042875	83.02914682	0.075251199	0.047726808
29	271	5.602118821	58.26055967	0.096156282	0.057194859
30	2	0.693147181	37.80589948	0.018334366	0.00252104
31	1	0	37.80589948	0	0
32	2776	7.928766322	44.12006804	0.179708842	0.127593141
33	3	1.098612289	44.12006804	0.024900512	0.004132395
34	11	2.397895273	110.6758025	0.02166594	0.006262861
35	63	4.143134726	50.98719439	0.081258339	0.038717193
36	1	0	27.41756364	0	0
37	24	3.17805383	27.41756364	0.115913065	0.043545899
38	341	5.831882477	61.36531734	0.095035481	0.057937418
39	73	4.290459441	59.50581288	0.072101518	0.035360145
40	4082	8.314342343	59.50581288	0.139723196	0.101065078
41	2	0.693147181	146.1230468	0.004743586	0.00065226
42	4038	8.303504799	121.5902694	0.068290866	0.049371928
43	29	3.36729583	116.0848847	0.029007186	0.011500214
44	126	4.836281907	72.87795312	0.066361385	0.035712991
45	139	4.934473933	65.50409114	0.075330775	0.041136777

46	2	0.693147181	65.50409114	0.010581739	0.001455026
47	1	0	83.91344318	0	0
48	60	4.094344562	81.45460816	0.050265352	0.023712856
49	40	3.688879454	63.10628787	0.058455022	0.025183468
50	24	3.17805383	29.54427129	0.107569207	0.040411302
51	200	5.298317367	29.54427129	0.179334847	0.102887938
52	13	2.564949357	39.43098218	0.065049086	0.019995884
53	8	2.079441542	31.08268764	0.066900313	0.017071505
54	16	2.772588722	31.08268764	0.089200418	0.029479135
55	4	1.386294361	123.2828768	0.011244825	0.002128148
56	36	3.583518938	79.88960117	0.044855887	0.018826948
57	164	5.099866428	58.2520937	0.087548208	0.048937408
58	679	6.520621128	58.2520937	0.11193797	0.07263608
59	54	3.988984047	72.27636303	0.055190714	0.025465443
60	301	5.707110265	57.94522597	0.098491466	0.059262642
61	39	3.663561646	46.86613174	0.078170771	0.033470138
62	1075	6.980075941	31.0238381	0.224990729	0.1510396
63	250	5.521460918	31.0238381	0.177974785	0.104894919
64	186	5.225746674	81.40692023	0.064192905	0.036488476
65	1	0	72.20414823	0	0
66	290	5.669880923	51.56199781	0.109962398	0.065898503
67	273	5.609471795	51.56199781	0.108790816	0.064763239
68	6	1.791759469	58.11601885	0.030830733	0.006976822
69	2	0.693147181	58.11601885	0.011926956	0.001639998
70	276	5.620400866	40.96980716	0.137183972	0.081765191
71	778	6.656726524	40.96980716	0.162478835	0.106557571
72	27	3.295836866	33.29937339	0.098975942	0.038467206
73	4	1.386294361	33.29937339	0.041631245	0.007878955
74	28865	10.27038507	99.03751647	0.103701965	0.080455488
75	13	2.564949357	71.73693893	0.035754932	0.010990954
76	4	1.386294361	61.73954208	0.022453914	0.004249534
77	1	0	45.41440353	0	0
78	37	3.610917913	36.28785066	0.099507627	0.042054194
79	163	5.093750201	36.28785066	0.140370678	0.078398399
80	4	1.386294361	57.63891176	0.024051363	0.00455186
81	27	3.295836866	57.63891176	0.057180761	0.022223422
82	2	0.693147181	62.24175615	0.011136369	0.00153129
83	2423	7.792761721	52.56292137	0.148255872	0.104520145
84	2	0.693147181	44.71214548	0.015502436	0.00213164
85	1338	7.198931241	34.63425846	0.207855793	0.141566481
86	2390	7.779048645	28.58435265	0.2721436	0.191721052
87	3113	8.04334217	28.58435265	0.281389691	0.200936648
88	2658	7.885329239	104.2134232	0.075665197	0.053602734
89	17	2.833213344	101.3244228	0.027961801	0.009430218
90	757	6.629363253	63.86283105	0.103806285	0.067936185
91	10	2.302585093	63.86283105	0.036055168	0.010050508
92	6	1.791759469	70.31951259	0.02548026	0.00576604
93	104	4.644390899	55.63986424	0.083472362	0.043580313
94	34	3.526360525	55.63986424	0.063378309	0.026215287

95	80	4.382026635	84.64517243	0.051769363	0.02582606
96	209	5.342334252	61.45684466	0.086928222	0.050147546
97	7	1.945910149	47.09654494	0.041317471	0.009979578
98	18	2.890371758	47.09654494	0.061371206	0.021089695
99	599	6.395261598	42.01777229	0.152203728	0.09775839
100	13	2.564949357	42.01777229	0.061044392	0.018764854
101	1636	7.400009517	48.64758104	0.152114645	0.104895464
102	142	4.955827058	48.64758104	0.101872014	0.055803283
103	68	4.219507705	79.45485195	0.053105727	0.025690317
104	3473	8.152774053	79.45485195	0.102608889	0.073661669
105	97	4.574710979	85.12619816	0.053740342	0.027733577
106	398	5.986452005	64.07010369	0.093435966	0.057846451
107	1222	7.10824414	64.07010369	0.110944789	0.075120794
108	7	1.945910149	70.26579216	0.027693563	0.006688939
109	4	1.386294361	55.25289739	0.025089985	0.004748425
110	5987	8.697345731	36.73914453	0.236732397	0.174099393
111	528	6.269096284	36.73914453	0.170638058	0.108424218
112	12	2.48490665	58.21149149	0.042687562	0.012745548
113	37	3.610917913	34.94601861	0.103328449	0.04366896
114	487	6.188264123	34.94601861	0.177080662	0.111714896
115	5	1.609437912	60.29531545	0.026692586	0.005580404
116	12352	9.421573272	36.80095787	0.256014349	0.193465522
117	4	1.386294361	30.9923162	0.04473026	0.008465462
118	3	1.098612289	30.9923162	0.035447892	0.005882799
119	4	1.386294361	154.3528154	0.008981335	0.00169977
120	2	0.693147181	111.7693475	0.006201586	0.00085274
121	1239	7.122059882	109.7820287	0.064874552	0.043966326
122	45	3.80666249	68.61241685	0.055480665	0.024578626
123	2	0.693147181	68.61241685	0.010102358	0.00138911
124	724	6.584791392	72.32745354	0.091041383	0.059376632
125	970	6.877296071	55.71808816	0.123430223	0.082270349
126	3820	8.248005702	55.71808816	0.148031025	0.10674763
127	202	5.308267697	141.4286622	0.037533182	0.021560503
128	5	1.609437912	102.0936565	0.015764328	0.003295721
129	10	2.302585093	81.51934263	0.028245874	0.007873639
130	2575	7.853604813	81.51934263	0.096340384	0.068137311
131	2	0.693147181	136.2758753	0.005086353	0.000699391
132	143	4.96284463	121.9616758	0.040691837	0.022312709
133	1	0	121.9616758	0	0
134	72	4.276666119	91.57653089	0.04670046	0.022842797
135	2	0.693147181	91.57653089	0.007569048	0.001040771
136	489	6.192362489	123.4400501	0.050164938	0.031659214
137	1	0	38.72539695	0	0
138	3	1.098612289	38.72539695	0.028369297	0.004708062
139	38	3.63758616	46.25916188	0.078634934	0.033454184
140	1392	7.238496841	46.25916188	0.156477043	0.106840597
141	1401	7.244941546	47.4416532	0.15271267	0.104312551
142	16	2.772588722	42.3710535	0.065435917	0.021625394
143	67	4.204692619	42.3710535	0.099235027	0.047866363

144	225	5.416100402	27.14706107	0.199509641	0.116135445
145	51	3.931825633	27.14706107	0.144834302	0.066001968
146	146	4.983606622	43.45275053	0.114690245	0.06307633
147	80	4.382026635	43.45275053	0.100845783	0.050308697
148	2283	7.733245647	48.73439127	0.158681486	0.111514576
149	1175	7.069023427	48.73439127	0.145052051	0.09796098
150	194	5.267858159	68.44719258	0.07696237	0.043984578
151	151	5.017279837	63.24196239	0.079334664	0.043840966
152	1	0	46.30557918	0	0
153	22	3.091042453	24.57317393	0.125789304	0.046042164
154	5	1.609437912	24.57317393	0.065495728	0.013692665
155	3	1.098612289	89.95950998	0.012212297	0.002026707
156	5	1.609437912	84.40816817	0.019067324	0.003986252
157	8	2.079441542	32.51759201	0.063948202	0.01631819
158	2	0.693147181	32.51759201	0.021316067	0.002931034
159	2936	7.98480339	53.1372465	0.150267541	0.106992352
160	11	2.397895273	45.37876872	0.052841788	0.015274702
161	4090	8.316300249	45.37876872	0.183264123	0.132571099
162	5	1.609437912	61.40540184	0.026210038	0.005479522
163	42	3.737669618	57.6842953	0.064795272	0.028244092
164	6	1.791759469	46.66530212	0.038395968	0.008688792
165	1	0	42.39172643	0	0
166	3586	8.184792654	35.28304796	0.231975215	0.166785879
167	8	2.079441542	33.95061745	0.061249005	0.015629414
168	88	4.477336814	28.29835622	0.158218971	0.080291797
169	645	6.469250317	23.21757353	0.278635935	0.180058534
170	3	1.098612289	23.21757353	0.047318135	0.00785274
171	165	5.105945474	47.15959015	0.108269505	0.060570293
172	20	2.995732274	36.016016	0.083177781	0.029562146
173	23	3.135494216	33.37533608	0.093946446	0.03485061
174	146	4.983606622	25.18836367	0.197853528	0.108813739
175	1	0	25.18836367	0	0
176	39	3.663561646	25.11076395	0.145896065	0.062467869
177	356	5.874930731	21.16456282	0.277583373	0.169968654
178	76	4.33073334	18.82949723	0.22999729	0.113654982
179	3839	8.252967195	18.82949723	0.438299923	0.316138219
180	24	3.17805383	90.11029867	0.035268486	0.013249567
181	131	4.875197323	69.20748984	0.070443204	0.038132539
182	396	5.981414211	38.30421674	0.156155502	0.0966291
183	336	5.81711116	38.30421674	0.151866078	0.092443068
184	1	0	40.06430664	0	0
185	4	1.386294361	40.06430664	0.034601731	0.006548579
186	1052	6.958448393	45.84845471	0.15177062	0.101734771
187	164	5.099866428	38.17756072	0.133582825	0.074669687
188	6	1.791759469	38.17756072	0.046932267	0.010620508
189	352	5.863631176	69.45681165	0.084421255	0.051633422
190	780	6.65929392	54.06187647	0.123179112	0.08079966
191	14	2.63905733	54.06187647	0.048815496	0.015406589
192	1393	7.239214974	59.1429713	0.122401949	0.083578317

193	784	6.66440902	53.01380031	0.125710833	0.082492447
194	3820	8.248005702	53.01380031	0.155582238	0.112192935
195	3	1.098612289	48.4131207	0.022692449	0.003765953
196	570	6.345636361	48.4131207	0.131072657	0.08383507
197	420	6.040254711	51.09676776	0.11821207	0.073563789
198	183	5.209486153	40.92741867	0.127285969	0.072198794
199	19	2.944438979	40.92741867	0.071942944	0.025157204
200	83	4.418840608	39.34976292	0.112296499	0.056396871
201	314	5.749392986	30.89706989	0.186082143	0.112472388
202	2	0.693147181	30.89706989	0.022434075	0.003084764
203	92	4.521788577	38.74572292	0.1167042	0.059684921
204	9	2.197224577	31.71964293	0.069270155	0.018530684
205	5240	8.564076777	31.71964293	0.269992849	0.197455179
206	3	1.098612289	21.52140876	0.051047415	0.008471637
207	487	6.188264123	21.52140876	0.287539919	0.181400338
208	73	4.290459441	72.8173673	0.058920826	0.028896048
209	54	3.988984047	72.8173673	0.054780668	0.025276245
210	5	1.609437912	52.89809041	0.030425255	0.006360763
211	4	1.386294361	39.47764401	0.035115934	0.006645895
212	747	6.616065185	39.47764401	0.167590173	0.109567235
213	13	2.564949357	52.99281432	0.048401833	0.014878571
214	15	2.708050201	46.40529147	0.058356496	0.018865709
215	2490	7.820037989	46.40529147	0.168516084	0.118974812
216	105	4.65396035	40.3541869	0.115327819	0.060306341
217	552	6.313548046	26.09851914	0.241912118	0.154305146
218	23600	10.06900199	18.7485456	0.537055098	0.41426137
219	63	4.143134726	18.7485456	0.220984327	0.105292489
220	108	4.682131227	44.88659997	0.104310222	0.054795615
221	11	2.397895273	39.35494311	0.060929964	0.01761271
222	25	3.218875825	36.20172252	0.088914991	0.033804343
223	12	2.48490665	36.20172252	0.068640564	0.020494532
224	94	4.543294782	68.97657865	0.06586721	0.033810664
225	119	4.779123493	54.35403377	0.087925829	0.046904434
226	8	2.079441542	54.35403377	0.038257355	0.009762445
227	35	3.555348061	56.5273999	0.062896013	0.026210378
228	340	5.828945618	41.4090794	0.140764917	0.085790046
229	1641	7.403061091	41.4090794	0.178778693	0.123304974
230	2	0.693147181	55.14896474	0.012568634	0.001728232
231	9	2.197224577	42.02321871	0.052285966	0.013987188
232	7	1.945910149	38.76819514	0.050193468	0.012123433
233	332	5.805134969	29.49326182	0.196829195	0.119664532
234	1705	7.44132039	26.71643709	0.278529669	0.192540644
235	15	2.708050201	23.11323333	0.11716449	0.037877381
236	5149	8.5465578	23.11323333	0.369769027	0.270222647
237	5	1.609437912	56.21409749	0.028630503	0.005985549
238	4	1.386294361	56.21409749	0.024660973	0.004667233
239	27	3.295836866	59.77041294	0.055141611	0.021430902
240	1	0	59.77041294	0	0
241	253	5.533389489	86.84515388	0.063715582	0.037604394

242	7	1.945910149	75.04243147	0.025930798	0.006263172
243	14259	9.565143565	56.05093645	0.170650915	0.129581947
244	2178	7.686162303	43.53689729	0.17654364	0.123750227
245	579	6.361302478	40.34720521	0.157664018	0.100977052
246	17	2.833213344	34.07473993	0.083147028	0.028041636
247	7038	8.859079318	34.07473993	0.259989639	0.192452597
248	4325	8.372167419	68.07149626	0.122990795	0.089195352
249	2695	7.899153483	37.46164995	0.210859732	0.149483601
250	4268	8.358900612	37.46164995	0.223132207	0.16172331
251	5	1.609437912	47.51365862	0.033873163	0.00708159
252	18	2.890371758	35.08539267	0.082381058	0.028309553
253	2	0.693147181	35.08539267	0.019756005	0.00271652
254	1	0	55.32507879	0	0
255	717	6.575075841	35.74838138	0.183926533	0.119864586
256	6	1.791759469	35.74838138	0.050121415	0.011342195
257	4	1.386294361	46.82677449	0.029604737	0.005602869
258	302	5.710427017	33.09403361	0.172551557	0.103861858
259	4074	8.312380597	33.09403361	0.251174598	0.181664232
260	3159	8.058010801	38.66758793	0.208391866	0.148917245
261	3032	8.016977747	36.031762	0.222497522	0.158675465
262	41	3.713572067	34.42610025	0.107870832	0.046750515
263	8444	9.041211409	29.43772786	0.30713007	0.228947412
264	341	5.831882477	26.8783969	0.216972854	0.132275302
265	2850	7.955074273	22.11300146	0.359746473	0.255760943
266	27	3.295836866	22.11300146	0.149045206	0.057926729
267	6	1.791759469	27.8604465	0.064311944	0.014553432
268	1109	7.011213987	27.8604465	0.25165476	0.169297773
269	935	6.840546529	24.07699975	0.284111251	0.188874906
270	47	3.850147602	24.07699975	0.159909775	0.071552378
271	31	3.433987204	25.57586631	0.134266701	0.054203222
272	329	5.796057751	21.35020187	0.271475548	0.164891219
273	7	1.945910149	21.35020187	0.091142471	0.022014013
274	392	5.97126184	25.91840832	0.230386904	0.142422956
275	118	4.770684624	21.69623831	0.219885335	0.117144823
276	6625	8.798605651	21.69623831	0.405535998	0.29947026
277	11	2.397895273	92.397749	0.02595188	0.007501776
278	39	3.663561646	80.5354586	0.045490045	0.019477333
279	21	3.044522438	63.45600354	0.047978477	0.017312976
280	135	4.905274778	60.00476455	0.081748088	0.044450274
281	3	1.098612289	50.30397822	0.021839471	0.003624396
282	35	3.555348061	50.30397822	0.070677274	0.029453029
283	27	3.295836866	58.32187766	0.056511158	0.021963179
284	388	5.96100534	43.89518444	0.135800895	0.083866858
285	1264	7.142036575	43.89518444	0.162706608	0.110411804
286	2161	7.678326357	64.60988223	0.118841361	0.083267407
287	2	0.693147181	50.46503923	0.013735196	0.001888638
288	1	0	41.17369306	0	0
289	3	1.098612289	30.46349668	0.036063237	0.005984919
290	169	5.129898715	30.46349668	0.168394941	0.094513065

291	1330	7.192934221	121.8333858	0.059039106	0.040195003
292	1855	7.525639975	96.7590816	0.077777092	0.054030018
293	21	3.044522438	74.33677564	0.040955804	0.014778853
294	29	3.36729583	74.33677564	0.045297846	0.017958824
295	731	6.59441346	90.68547453	0.072717417	0.047461461
296	1172	7.06646697	57.36727515	0.123179408	0.08317515
297	854	6.749931194	57.36727515	0.117661701	0.077706839
298	6212	8.734238184	64.54022351	0.135330151	0.099675838
299	267	5.587248658	64.54022351	0.086570023	0.051406946
300	7717	8.951180966	55.70918397	0.160676936	0.119365623
301	10	2.302585093	29.63617172	0.077695092	0.021657787
302	1	0	21.59023683	0	0
303	90	4.49980967	21.59023683	0.208418727	0.106183863
304	2	0.693147181	60.56477709	0.011444724	0.00157369
305	2	0.693147181	41.98187106	0.016510631	0.00227027
306	3	1.098612289	41.98187106	0.026168731	0.004342864
307	52	3.951243719	27.40961875	0.14415537	0.065972781
308	1	0	20.30628641	0	0
309	11	2.397895273	17.33899053	0.138294976	0.039976213
310	9	2.197224577	17.33899053	0.126721597	0.033899705
311	61	4.110873864	108.2001612	0.037993232	0.017984355
312	5	1.609437912	70.4687758	0.022839022	0.004774771
313	17	2.833213344	66.33263859	0.042712206	0.014404846
314	9	2.197224577	58.84244515	0.03734081	0.009989161
315	1414	7.254177846	51.69179354	0.140335193	0.095913435
316	804	6.689599269	51.69179354	0.129413178	0.085084028
317	2582	7.856319571	61.72184359	0.127285886	0.090036424
318	846	6.74051936	51.86289514	0.12996805	0.085774548
319	2649	7.881937489	46.26815691	0.170353392	0.120660612
320	177	5.176149733	46.26815691	0.111872832	0.063178691
321	2	0.693147181	86.0878453	0.008051627	0.001107127
322	23	3.135494216	75.70680712	0.041416279	0.015363887
323	6	1.791759469	75.70680712	0.023667085	0.005355729
324	8	2.079441542	71.5154189	0.029076828	0.007419774
325	1189	7.080867897	67.31784726	0.105185596	0.07109294
326	6711	8.81150325	56.57416497	0.155751362	0.115074758
327	12	2.48490665	39.74401226	0.062522793	0.018667902
328	4	1.386294361	39.74401226	0.034880584	0.006601353
329	22	3.091042453	60.36405779	0.051206671	0.018742976
330	25	3.218875825	55.24376474	0.058266772	0.022152282
331	413	6.023447593	35.31651188	0.17055613	0.105968003
332	73	4.290459441	25.99998577	0.165017761	0.080928281
333	472	6.156978986	25.99998577	0.236807014	0.148972473
334	2	0.693147181	36.70208877	0.018885769	0.00259686
335	123	4.812184355	36.70208877	0.131114727	0.070301634
336	52	3.951243719	55.60565231	0.071058311	0.032519873
337	36	3.583518938	55.60565231	0.064445228	0.027049002
338	1	0	69.17505053	0	0
339	12	2.48490665	67.78398342	0.036659201	0.010945614

340	2	0.693147181	53.92462586	0.012854001	0.00176747
341	4	1.386294361	29.09009266	0.047655206	0.009019025
342	19	2.944438979	29.09009266	0.101217931	0.035394161
343	1	0	57.32766188	0	0
344	23	3.135494216	35.56950704	0.088151186	0.032700785
345	102	4.624972813	35.56950704	0.13002634	0.067668779
346	2	0.693147181	46.02151982	0.015061371	0.002070992
347	1	0	42.42999797	0	0
348	565	6.336825731	30.37465458	0.20862215	0.133336308
349	331	5.802118375	22.12653817	0.262224408	0.159372447
350	4901	8.497194545	22.12653817	0.384027292	0.280045802
351	385	5.953243334	72.15595889	0.082505221	0.05091424
352	26	3.258096538	72.15595889	0.045153534	0.017361878
353	7	1.945910149	64.66495955	0.030092188	0.007268289
354	19400	9.873028345	59.36417161	0.166312914	0.127533272
355	7	1.945910149	52.06108713	0.03737744	0.009027926
356	1911	7.555381944	52.06108713	0.14512532	0.100987045
357	1	0	94.19025976	0	0
358	11742	9.370927437	83.26822135	0.112539061	0.084895635
359	1	0	59.74829742	0	0
360	130	4.86753445	57.78109636	0.08424095	0.045549306
361	1470	7.29301768	51.96651307	0.140340717	0.096149156
362	2	0.693147181	51.96651307	0.013338343	0.001834069
363	114	4.736198448	56.54550558	0.083759061	0.04438194
364	134	4.8978398	44.20983027	0.110786216	0.060173485
365	1448	7.277938573	34.52038815	0.210830149	0.144307465
366	2437	7.798523054	34.52038815	0.225910642	0.159315249
367	16	2.772588722	38.66157524	0.071714324	0.023700295
368	5	1.609437912	38.66157524	0.041628876	0.008703014
369	1357	7.21303166	52.84296866	0.136499365	0.093050354
370	3	1.098612289	40.80701748	0.026922141	0.004467897
371	1738	7.460490306	36.02418333	0.207096723	0.143322339
372	2	0.693147181	36.02418333	0.019241163	0.002645728
373	30	3.401197382	23.47978585	0.144856406	0.057963755
374	45	3.80666249	23.47978585	0.162125094	0.071823438
375	41	3.713572067	80.68147562	0.046027568	0.019948048
376	1162	7.057897937	58.01908573	0.121647865	0.082094163
377	93	4.532599493	28.53900197	0.158821233	0.081375926
378	154	5.036952602	28.53900197	0.176493649	0.097801777
379	93	4.532599493	32.80662519	0.138161102	0.070790205
380	1	0	22.48036981	0	0
381	264	5.575949103	22.48036981	0.24803636	0.147101081
382	2	0.693147181	61.49658681	0.011271311	0.001549845
383	1770	7.478734826	61.49658681	0.121612194	0.084252182
384	6	1.791759469	98.37378718	0.01821379	0.004121678
385	355	5.872117789	36.87783781	0.159231618	0.09747233
386	871	6.769641977	36.87783781	0.183569384	0.12140996
387	450	6.109247583	47.42032316	0.128831842	0.080692515
388	826	6.716594774	42.26728324	0.158907653	0.104687273

389	66	4.189654742	38.12295386	0.109898482	0.052852752
390	1	0	38.12295386	0	0
391	2	0.693147181	93.98401306	0.00737516	0.001014111
392	290	5.669880923	59.47115289	0.095338339	0.057134566
393	185	5.220355825	59.47115289	0.08777963	0.049860696
394	1621	7.390798522	66.94787269	0.110396316	0.076085318
395	70	4.248495242	66.94787269	0.06345975	0.030872717
396	391	5.96870756	94.91358957	0.0628857	0.038865662
397	50	3.912023005	71.30645572	0.054862115	0.024891889
398	1301	7.170888479	71.30645572	0.100564366	0.068369649
399	246	5.505331536	87.05739757	0.063237952	0.037201646
400	739	6.605297921	59.63795857	0.110756607	0.072350194
401	403	5.998936562	59.63795857	0.100589234	0.062350193
402	10	2.302585093	84.33848877	0.027301712	0.00761045
403	6742	8.816111896	29.39891229	0.299878846	0.221602104
404	37	3.610917913	29.39891229	0.122824881	0.051908597
405	2171	7.68294317	68.98940841	0.111364097	0.078048141
406	101	4.615120517	68.98940841	0.066896073	0.034757441
407	9	2.197224577	64.9310724	0.033839339	0.009052471
408	42	3.737669618	39.22176812	0.095295796	0.041539192
409	215	5.370638028	39.22176812	0.136930034	0.079268761
410	546	6.302618976	31.29664505	0.201383214	0.128332702
411	5	1.609437912	31.29664505	0.051425254	0.010751064
412	220	5.393627546	49.82199646	0.108257957	0.062846476
413	2	0.693147181	49.82199646	0.013912473	0.001913014
414	19	2.944438979	60.41071379	0.048740344	0.017043656
415	75	4.317488114	60.41071379	0.071468914	0.035229375
416	11	2.397895273	67.84910246	0.035341592	0.010216011
417	27	3.295836866	67.84910246	0.048575983	0.018879157
418	1033	6.940222469	72.31582925	0.095971	0.064250278
419	46	3.828641396	65.71780396	0.058258815	0.02594043
420	203	5.313205979	59.30228081	0.089595306	0.051498882
421	1780	7.484368643	46.71479691	0.160214089	0.111031779
422	1217	7.104144093	46.71479691	0.152074815	0.102942265
423	2	0.693147181	55.58551727	0.012469924	0.001714659
424	17	2.833213344	42.77816192	0.066230367	0.022336431
425	2181	7.687538766	42.77816192	0.179707085	0.125977169
426	367831	12.81537887	NA	NA	NA
427	1152	7.049254841	326.6565196	0.021580022	0.014554897
428	322	5.774551546	292.8599401	0.019717793	0.011949512
429	830	6.721425701	292.8599401	0.02295099	0.015125406
430	829	6.720220155	270.6661112	0.024828451	0.016361239
431	117	4.762173935	226.9311016	0.020985109	0.011165049
432	46	3.828641396	147.0862728	0.026029903	0.011590124
433	712	6.568077911	226.9311016	0.028943049	0.018851775
434	446	6.100318952	171.1536964	0.035642344	0.022305754
435	240	5.480638923	125.4437612	0.043690008	0.025627961
436	206	5.327876169	125.4437612	0.042472229	0.024457597
437	205	5.323009979	101.4737174	0.052457031	0.030189009

438	366679	12.81224208	326.6565196	0.039222368	0.032173494
439	366678	12.81223936	183.9906694	0.069635267	0.057120716
440	171	5.141663557	178.132937	0.028864193	0.016225925
441	159	5.068904202	108.2887884	0.046809132	0.02605421
442	366507	12.8117729	178.132937	0.071922538	0.058996458
443	91	4.510859507	169.526009	0.026608657	0.013582489
444	90	4.49980967	95.85730438	0.046942794	0.02391612
445	366416	12.81152458	169.526009	0.07557262	0.061990276
446	13058	9.477156252	160.7745018	0.058946886	0.044629342
447	12974	9.470702634	156.9385143	0.06034658	0.045679106
448	4809	8.478244441	133.6778177	0.063422972	0.046212073
449	93	4.532599493	124.7712083	0.036327287	0.01861317
450	4716	8.458716262	124.7712083	0.067793815	0.049354637
451	746	6.6147256	102.6728017	0.064425296	0.042115659
452	745	6.613384218	71.56365766	0.092412608	0.060405063
453	3970	8.286521374	102.6728017	0.080708048	0.058303666
454	8165	9.007612005	133.6778177	0.067382997	0.050166353
455	3570	8.180320875	124.2160072	0.06585561	0.047338936
456	3053	8.023879993	83.02914682	0.096639316	0.068942518
457	2782	7.930925372	58.26055967	0.136128548	0.096661793
458	3	1.098612289	55.88455912	0.019658602	0.003262467
459	2779	7.92984643	55.88455912	0.141896913	0.100752243
460	4595	8.432724035	124.2160072	0.067887579	0.049366389
461	4584	8.430327258	110.6758025	0.076171368	0.055384316
462	88	4.477336814	75.14568721	0.059582086	0.030236278
463	25	3.218875825	50.98719439	0.063131064	0.024001623
464	4496	8.410943392	75.14568721	0.111928491	0.08131349
465	4155	8.332067707	61.36531734	0.135778125	0.098290803
466	353358	12.77523699	160.7745018	0.079460591	0.06513892
467	78499	11.27084116	159.715531	0.070568223	0.056152152
468	78497	11.27081569	146.1230468	0.077132362	0.061375296
469	4696	8.454466362	135.5066609	0.062391519	0.045413236
470	658	6.489204931	121.5902694	0.053369443	0.034543923
471	629	6.444131257	116.0848847	0.055512234	0.035799262
472	267	5.587248658	94.63660583	0.05903898	0.035058482
473	141	4.94875989	72.87795312	0.06790476	0.037158703
474	362	5.891644212	94.63660583	0.062255447	0.038184135
475	361	5.888877958	83.91344318	0.070178004	0.043031459
476	301	5.707110265	81.45460816	0.070064916	0.042158293
477	261	5.564520407	63.10628787	0.088176957	0.052226759
478	224	5.411646052	56.68156248	0.095474539	0.05554634
479	37	3.610917913	56.68156248	0.063705335	0.026923328
480	24	3.17805383	39.43098218	0.080597887	0.030278791
481	73801	11.20912756	135.5066609	0.082720122	0.06572861
482	73797	11.20907336	123.2828768	0.090921575	0.072245315
483	2598	7.862497197	118.1310528	0.066557412	0.047094902
484	879	6.778784898	92.92772551	0.07294685	0.048278236
485	843	6.736966958	79.88960117	0.084328459	0.055639299
486	1719	7.449498005	92.92772551	0.080164428	0.055442386

487	1665	7.417580402	72.27636303	0.102628025	0.070844546
488	1364	7.218176838	57.94522597	0.124568965	0.084945191
489	1325	7.189167738	46.86613174	0.153397933	0.104411266
490	71199	11.17323405	118.1310528	0.094583378	0.075092663
491	1843	7.519149958	115.5037443	0.065098755	0.045205775
492	1812	7.502186487	96.58717913	0.077672695	0.053884543
493	750	6.620073207	87.01980565	0.076075477	0.049752084
494	564	6.335054251	81.40692023	0.077819603	0.049729195
495	563	6.333279628	72.20414823	0.087713515	0.056043233
496	1062	6.967909202	87.01980565	0.080072682	0.053709187
497	8	2.079441542	62.55057844	0.033244162	0.008483187
498	1054	6.960347729	62.55057844	0.111275513	0.074599874
499	31	3.433987204	96.58717913	0.03555324	0.014352778
500	69356	11.14700794	115.5037443	0.096507763	0.076573731
501	38382	10.55534388	112.0012477	0.094243092	0.073686619
502	9517	9.160834952	99.03751647	0.092498634	0.069258553
503	218	5.384495063	85.72188701	0.062813539	0.036424361
504	205	5.323009979	71.73693893	0.0742018	0.042703117
505	201	5.303304908	61.73954208	0.085898028	0.049312359
506	200	5.298317367	45.41440353	0.116666012	0.066933592
507	9299	9.137662146	85.72188701	0.106596605	0.07974678
508	31	3.433987204	65.24259678	0.052634128	0.021248301
509	9268	9.134322886	65.24259678	0.140005508	0.10472772
510	9266	9.134107066	62.24175615	0.146752078	0.109773458
511	6843	8.830981511	52.56292137	0.168007814	0.124226557
512	6841	8.830689199	44.71214548	0.197500905	0.146032331
513	5503	8.613048677	34.63425846	0.248685812	0.182250119
514	30974	10.34090342	112.0012477	0.092328466	0.071772494
515	6290	8.74671635	106.6664132	0.082000661	0.060427279
516	3632	8.19753874	104.2134232	0.078661064	0.056589913
517	3615	8.192847135	101.3244228	0.080857575	0.058157238
518	911	6.814542897	93.73385123	0.072700981	0.048240721
519	767	6.642486801	76.13363142	0.087247734	0.057156967
520	144	4.9698133	76.13363142	0.065277502	0.035829801
521	138	4.927253685	70.31951259	0.070069508	0.038223352
522	2704	7.902487437	93.73385123	0.084307722	0.059778033
523	2624	7.87245515	84.64517243	0.093005365	0.065843023
524	234	5.455321115	74.24830709	0.073474013	0.042970358
525	25	3.218875825	61.45684466	0.052376197	0.019912761
526	2390	7.779048645	74.24830709	0.104770721	0.073809389
527	612	6.416732283	57.54179965	0.111514279	0.071752118
528	1778	7.483244416	57.54179965	0.130048842	0.09012072
529	24684	10.11391054	106.6664132	0.094818137	0.073234768
530	3541	8.172164452	90.28384342	0.090516355	0.065040627
531	21143	9.95906416	90.28384342	0.110308376	0.084809246
532	21046	9.954465797	85.12619816	0.116937747	0.089893692
533	8146	9.005282288	81.88874469	0.10996972	0.081864748
534	1620	7.390181428	75.20982402	0.098260853	0.067719032
535	6526	8.783549477	75.20982402	0.116787263	0.086190104

536	6519	8.782476269	70.26579216	0.124989358	0.092239347
537	6515	8.78186249	55.25289739	0.158939402	0.117290824
538	12900	9.46498259	81.88874469	0.115583437	0.087473498
539	536	6.284134161	69.84971916	0.089966491	0.05724004
540	524	6.261491684	58.21149149	0.107564529	0.068301571
541	12364	9.422544303	69.84971916	0.134897383	0.101942956
542	12359	9.422139822	60.29531545	0.156266532	0.118090147
543	7	1.945910149	36.80095787	0.052876617	0.012771505
544	274859	12.52401352	159.715531	0.0784145	0.063997916
545	274855	12.52399896	154.3528154	0.081138779	0.066221316
546	6802	8.824971966	145.450314	0.060673447	0.044851805
547	6800	8.824677891	111.7693475	0.078954365	0.058364978
548	5561	8.623533227	109.7820287	0.078551411	0.057591988
549	47	3.850147602	86.20719521	0.044661557	0.019984023
550	5514	8.615045592	86.20719521	0.099934183	0.073243206
551	4790	8.47428569	72.32745354	0.117165547	0.08535594
552	268053	12.49894	145.450314	0.085932712	0.07010221
553	267851	12.49818613	141.4286622	0.088370956	0.0720903
554	2590	7.859413155	139.9728021	0.056149574	0.039724124
555	2585	7.857480787	102.0936565	0.076963457	0.054443846
556	265261	12.48846953	139.9728021	0.089220687	0.072770697
557	265259	12.48846199	136.2758753	0.091641033	0.074744784
558	144	4.9698133	134.1260727	0.037053298	0.020337976
559	265115	12.48791897	134.1260727	0.093105827	0.075938761
560	74	4.304065093	128.6151585	0.03346468	0.016454169
561	265041	12.48763981	128.6151585	0.097093064	0.079190422
562	146137	11.89229982	125.8278758	0.094512442	0.076213448
563	145648	11.88894803	123.4400501	0.096313539	0.077660571
564	4	1.386294361	120.3972274	0.011514338	0.002179155
565	145644	11.88892057	120.3972274	0.098747461	0.079623073
566	3190	8.067776196	117.4945866	0.068665089	0.049091695
567	2914	7.977281987	72.04143866	0.110731853	0.078812705
568	1430	7.265429723	58.05093377	0.125156122	0.085599288
569	1484	7.302496424	58.05093377	0.125794642	0.086233888
570	83	4.418840608	47.4416532	0.093142635	0.046777533
571	276	5.620400866	72.04143866	0.078016222	0.046499683
572	142454	11.86677442	117.4945866	0.100998478	0.081401644
573	20161	9.911505324	114.4614213	0.086592541	0.066479749
574	4057	8.308199063	94.50160081	0.087915961	0.063573843
575	3684	8.211754397	84.74361255	0.09690116	0.06975876
576	226	5.420534999	79.88704896	0.067852488	0.039518301
577	3458	8.148445666	79.88704896	0.101999583	0.073209111
578	373	5.92157842	84.74361255	0.069876398	0.04298655
579	179	5.187385806	68.44719258	0.075786685	0.042863071
580	28	3.33220451	63.24196239	0.052689771	0.020687733
581	27	3.295836866	46.30557918	0.071175805	0.027662624
582	16104	9.686822967	94.50160081	0.102504327	0.078144672
583	16101	9.686636661	89.95950998	0.107677739	0.082088157
584	16096	9.686326073	84.40816817	0.114755791	0.087483239

585	10	2.302585093	76.63545778	0.030045949	0.008375417
586	16086	9.685704607	76.63545778	0.126386726	0.096348075
587	7037	8.858937222	66.4812136	0.133254746	0.098638847
588	4101	8.318986125	53.1372465	0.15655659	0.113265056
589	9049	9.110409533	66.4812136	0.137037353	0.102417182
590	9044	9.109856833	61.40540184	0.148355952	0.110874063
591	9002	9.105202054	57.6842953	0.157845424	0.117945729
592	4337	8.374938144	54.92455826	0.152480756	0.110595811
593	4331	8.373553741	46.66530212	0.179438541	0.130140474
594	4330	8.373322821	42.39172643	0.197522572	0.143254701
595	744	6.612041035	35.28304796	0.187399939	0.122480352
596	736	6.601230119	33.95061745	0.194436232	0.126972628
597	648	6.473890696	28.29835622	0.228772677	0.147891944
598	4665	8.447843113	54.92455826	0.153808121	0.111920525
599	4500	8.411832676	47.15959015	0.178369503	0.129586486
600	4480	8.407378325	36.016016	0.233434434	0.169557903
601	4457	8.402231173	33.37533608	0.25174971	0.182819533
602	147	4.990432587	31.22232049	0.159835416	0.087990606
603	4310	8.368693183	31.22232049	0.268035593	0.194354359
604	4271	8.359603271	25.11076395	0.332909157	0.241295853
605	3915	8.272570608	21.16456282	0.390868958	0.28218309
606	122293	11.71417508	114.4614213	0.102341688	0.08222564
607	892	6.793466133	106.0460973	0.064061444	0.042443053
608	868	6.766191715	90.11029867	0.075087885	0.049649396
609	737	6.602587892	69.20748984	0.095402794	0.062307425
610	732	6.595780514	65.20287884	0.101157811	0.066031065
611	5	1.609437912	65.20287884	0.024683541	0.005160389
612	121401	11.70685439	106.0460973	0.110394014	0.088681655
613	15790	9.667132107	104.2608699	0.092720616	0.070641237
614	1222	7.10824414	92.97176657	0.076455944	0.051768372
615	170	5.135798437	45.84845471	0.112016827	0.062920348
616	14568	9.586582621	92.97176657	0.103112837	0.078352982
617	14216	9.56212337	69.45681165	0.137670059	0.10452785
618	794	6.677083461	66.3354971	0.100656266	0.066114973
619	13422	9.504650431	66.3354971	0.143281514	0.108580413
620	5997	8.699014623	63.04143767	0.137988836	0.101487678
621	4604	8.43468077	59.1429713	0.1426151	0.103715597
622	7425	8.912607964	63.04143767	0.141376978	0.104871248
623	573	6.350885717	61.23734472	0.103709358	0.06636286
624	6852	8.832295859	61.23734472	0.144230549	0.106650989
625	622	6.432940093	56.61872556	0.113618596	0.073204063
626	202	5.308267697	51.09676776	0.103886565	0.059676437
627	6230	8.737131612	56.61872556	0.154315229	0.113672465
628	5740	8.655214489	54.24032064	0.159571595	0.117148941
629	399	5.988961417	46.58690261	0.128554617	0.079607827
630	316	5.755742214	39.34976292	0.146271331	0.08846915
631	5341	8.58316818	46.58690261	0.184239941	0.134850492
632	5249	8.565792861	38.74572292	0.221077121	0.161693225
633	490	6.194405391	54.24032064	0.114202964	0.072086982

634	105611	11.56751781	104.2608699	0.110947835	0.088863808
635	37333	10.52763293	97.52301074	0.107950245	0.084342032
636	883	6.783325201	92.49744654	0.07333527	0.048551406
637	127	4.844187086	86.57352478	0.055954602	0.030148591
638	756	6.628041376	86.57352478	0.07655968	0.050099505
639	751	6.621405652	52.89809041	0.125172867	0.08186937
640	36450	10.50369674	92.49744654	0.113556613	0.088665783
641	26994	10.2033699	76.03592689	0.134191432	0.103912959
642	2518	7.831220215	58.77209391	0.133247256	0.094129759
643	2505	7.826044014	52.99281432	0.14768123	0.104298014
644	24476	10.10544832	58.77209391	0.171942969	0.13277102
645	24320	10.09905434	49.31506659	0.204786387	0.15810258
646	24215	10.09472755	40.3541869	0.250153165	0.193102988
647	23663	10.07166793	26.09851914	0.385909556	0.297697469
648	156	5.049856007	49.31506659	0.102399862	0.056845921
649	48	3.871201011	44.88659997	0.086244024	0.038774738
650	37	3.610917913	39.35494311	0.091752589	0.038776738
651	9456	9.15440474	76.03592689	0.12039578	0.090125435
652	9362	9.144414222	68.97657865	0.132572743	0.099204544
653	127	4.844187086	65.51146834	0.073944108	0.039841418
654	9235	9.130755893	65.51146834	0.13937645	0.104243502
655	2016	7.608870629	62.44676416	0.121845715	0.085044277
656	1981	7.591357047	56.5273999	0.134295175	0.093641399
657	7219	8.884471718	62.44676416	0.142272732	0.105419915
658	7217	8.884194633	55.14896474	0.161094495	0.119364994
659	7208	8.882946799	42.02321871	0.211381876	0.156618406
660	7201	8.881975184	38.76819514	0.229104686	0.169743242
661	6869	8.834773814	29.49326182	0.29955228	0.221525111
662	5164	8.549466752	26.71643709	0.320007744	0.233886837
663	68278	11.13134289	97.52301074	0.114140681	0.090531348
664	37	3.610917913	89.55229841	0.04032189	0.017040951
665	9	2.197224577	86.81620697	0.025308922	0.006770472
666	28	3.33220451	86.81620697	0.038382286	0.015070145
667	68241	11.13080084	89.55229841	0.12429386	0.098583149
668	67988	11.1270865	86.84515388	0.128125589	0.101613428
669	67981	11.12698353	75.04243147	0.148275893	0.117593882
670	24071	10.08876308	71.59311379	0.140918065	0.108761184
671	9812	9.191361405	56.05093645	0.163982299	0.12291843
672	7634	8.940367233	43.53689729	0.205351502	0.152490434
673	7055	8.861491864	40.34720521	0.219630872	0.162593211
674	43910	10.68989736	71.59311379	0.149314603	0.117155363
675	39585	10.58620554	68.07149626	0.155515981	0.121693341
676	6988	8.851949671	66.53044703	0.133051108	0.09846096
677	6963	8.848365695	51.81820194	0.170757868	0.12634696
678	25	3.218875825	51.81820194	0.062118632	0.02361671
679	20	2.995732274	47.51365862	0.063049918	0.022408519
680	32597	10.39197554	66.53044703	0.156198793	0.121593449
681	32596	10.39194486	55.32507879	0.187834253	0.146220051
682	723	6.583409222	50.95569067	0.129198705	0.084253503

683	31873	10.36951454	50.95569067	0.203500618	0.158318171
684	31869	10.36938903	46.82677449	0.221441454	0.17227508
685	4376	8.383890344	40.0345209	0.209416527	0.151952857
686	27493	10.22168671	40.0345209	0.255321819	0.197815004
687	24334	10.09962983	38.66758793	0.2611911	0.201652468
688	21302	9.966556244	36.031762	0.276604742	0.21271215
689	21261	9.964629687	34.42610025	0.289449854	0.222577282
690	11662	9.364090972	32.62759722	0.286999098	0.216451039
691	3218	8.076515328	29.43772786	0.274359331	0.196235359
692	2877	7.964503364	26.8783969	0.29631616	0.210765607
693	9599	9.169414205	32.62759722	0.281032469	0.210489489
694	1115	7.016609684	30.69062766	0.228623857	0.153860129
695	8484	9.045937316	30.69062766	0.294745921	0.219754791
696	982	6.889591308	29.61166416	0.232664779	0.15521348
697	7502	8.922924931	29.61166416	0.301331424	0.223612518
698	367	5.905361848	28.47748278	0.207369517	0.127363927
699	336	5.81711116	25.57586631	0.227445322	0.138449243
700	7135	8.87276753	28.47748278	0.311571342	0.230759266
701	6743	8.816260209	25.91840832	0.340154384	0.251366089
702	118904	11.68607172	125.8278758	0.092873472	0.074574591
703	4259	8.35678967	123.5674879	0.067629356	0.049012209
704	4248	8.354203563	92.397749	0.090415661	0.065518207
705	233	5.451038454	83.81818225	0.065034081	0.038015053
706	194	5.267858159	80.5354586	0.06541042	0.037382551
707	173	5.153291594	63.45600354	0.081210466	0.045723358
708	38	3.63758616	60.00476455	0.060621622	0.02579066
709	4015	8.297792626	83.81818225	0.098997525	0.07155305
710	1679	7.425953657	79.86540095	0.09298086	0.064216977
711	1652	7.409741954	58.32187766	0.127049098	0.087661616
712	2336	7.756195344	79.86540095	0.097115838	0.068333165
713	175	5.164785974	64.60988223	0.079938019	0.045075932
714	173	5.153291594	50.46503923	0.102116072	0.057493695
715	172	5.147494477	41.17369306	0.125019013	0.070334034
716	114645	11.64959568	123.5674879	0.094277191	0.075643596
717	113315	11.63792683	121.8333858	0.095523298	0.076624491
718	42740	10.66289053	117.3133986	0.09089235	0.071266506
719	18959	9.850034032	114.7542296	0.085835913	0.065774687
720	1905	7.552237288	111.8225425	0.0675377	0.046988428
721	50	3.912023005	96.7590816	0.040430551	0.018344039
722	17054	9.744140059	111.8225425	0.087139318	0.066552614
723	16323	9.700330435	90.68547453	0.106966749	0.081581936
724	2026	7.613818685	89.20845746	0.085348619	0.059587019
725	14297	9.567805004	89.20845746	0.107252219	0.081447986
726	6479	8.776321456	83.25710909	0.105412277	0.077772632
727	7818	8.964184046	83.25710909	0.107668692	0.080026193
728	101	4.615120517	55.70918397	0.082843082	0.043043087
729	91	4.510859507	29.63617172	0.152207902	0.077695092
730	23781	10.07664222	114.7542296	0.087810639	0.067748575
731	80	4.382026635	111.702544	0.039229426	0.019570291

732	7	1.945910149	81.85140441	0.023773693	0.005742157
733	5	1.609437912	60.56477709	0.026573827	0.005555576
734	73	4.290459441	81.85140441	0.052417664	0.025706757
735	21	3.044522438	27.40961875	0.111074965	0.040081269
736	20	2.995732274	20.30628641	0.147527333	0.052432568
737	23701	10.07327252	111.702544	0.090179437	0.069569294
738	23640	10.07069547	108.2001612	0.093074681	0.071797407
739	8503	9.048174321	101.6917605	0.088976474	0.066344088
740	8498	9.047586121	70.4687758	0.128391419	0.095731187
741	8481	9.045583646	66.33263859	0.136367011	0.101670299
742	2227	7.708410667	63.34046658	0.12169804	0.085409203
743	2218	7.704361168	58.84244515	0.130932036	0.091869493
744	6254	8.740976538	63.34046658	0.137999876	0.101670067
745	3672	8.208491752	61.72184359	0.132991681	0.095725505
746	2826	7.946617563	51.86289514	0.153223563	0.108887329
747	15137	9.624897357	101.6917605	0.09464776	0.072010816
748	31	3.433987204	95.99419293	0.035772864	0.01444144
749	29	3.36729583	86.0878453	0.039114649	0.015507428
750	15106	9.622847295	95.99419293	0.100244056	0.076263549
751	8929	9.097059686	90.49437905	0.10052624	0.075092863
752	8921	9.096163327	71.5154189	0.127191639	0.095008694
753	7732	8.95312284	67.31784726	0.132997759	0.098810365
754	6727	8.813884558	63.34412155	0.139142897	0.102813589
755	16	2.772588722	56.57416497	0.049008036	0.016196275
756	1005	6.91274282	63.34412155	0.109129982	0.072920312
757	983	6.89060912	60.36405779	0.11415086	0.076156875
758	958	6.864847778	55.24376474	0.124264662	0.082753473
759	545	6.300785795	35.31651188	0.178409063	0.113674295
760	6177	8.728587996	90.49437905	0.096454477	0.071026056
761	125	4.828313737	79.43392478	0.060784026	0.032671868
762	6052	8.708144075	79.43392478	0.109627519	0.080658799
763	88	4.477336814	77.66028148	0.057652853	0.029257245
764	5964	8.693496676	77.66028148	0.111942637	0.082312598
765	5963	8.693328989	69.17505053	0.125671451	0.092406901
766	5951	8.691314552	67.78398342	0.12822077	0.094273608
767	25	3.218875825	61.24427193	0.05255799	0.019981876
768	23	3.135494216	53.92462586	0.058145869	0.021569938
769	5926	8.687104728	61.24427193	0.141843546	0.104271583
770	5925	8.686935966	57.32766188	0.151531315	0.11139245
771	125	4.828313737	49.61364876	0.097318255	0.052309289
772	5800	8.665613197	49.61364876	0.174661881	0.128282817
773	5798	8.665268309	46.02151982	0.188287313	0.138288225
774	5797	8.665095821	42.42999797	0.204220981	0.149989686
775	5232	8.562548893	30.37465458	0.28189782	0.20614827
776	70575	11.16443125	117.3133986	0.095167572	0.075541019
777	411	6.018593214	113.4596728	0.053046101	0.032942715
778	70164	11.15859064	113.4596728	0.098348518	0.078055344
779	43808	10.68757173	111.6017071	0.095765307	0.07513498
780	21325	9.967635372	103.4838132	0.09632072	0.074074118

781	21318	9.967307065	64.66495955	0.154137683	0.118536285
782	1918	7.559038255	59.36417161	0.12733334	0.088624745
783	22483	10.02051475	103.4838132	0.096831712	0.0745849
784	17479	9.768755439	101.4882165	0.096255071	0.073571941
785	17478	9.768698226	94.19025976	0.103712404	0.079271763
786	5736	8.654517382	83.26822135	0.103935418	0.076301619
787	1603	7.379632153	68.49198814	0.107744458	0.074207889
788	1602	7.379008128	59.74829742	0.123501563	0.08505724
789	1472	7.294377299	57.78109636	0.126241587	0.086496935
790	4133	8.326758815	68.49198814	0.121572742	0.087986188
791	4019	8.298788394	56.54550558	0.146763006	0.106081643
792	3885	8.264878263	44.20983027	0.186946618	0.134915856
793	5004	8.517992872	101.4882165	0.083930856	0.06126036
794	3196	8.069655307	97.97473175	0.082364658	0.058891534
795	3121	8.045908742	60.26012798	0.133519609	0.095356638
796	21	3.044522438	55.82175002	0.054540075	0.019680721
797	3100	8.03915739	55.82175002	0.144014786	0.102817832
798	1743	7.463363046	52.84296866	0.141236634	0.097759992
799	1740	7.461640392	40.80701748	0.182851893	0.126552115
800	75	4.317488114	60.26012798	0.07164751	0.035317411
801	1808	7.499976541	97.97473175	0.07655011	0.053098966
802	1767	7.477038472	80.68147562	0.092673546	0.064197312
803	605	6.405228458	58.01908573	0.110398645	0.070966472
804	247	5.509388337	42.02562963	0.131095914	0.077157496
805	358	5.880532986	42.02562963	0.139927302	0.085728085
806	265	5.579729826	32.80662519	0.17007936	0.100910807
807	26356	10.17945123	111.6017071	0.091212325	0.070583217
808	1772	7.479864131	107.4858296	0.069589305	0.048214218
809	24584	10.1098511	107.4858296	0.094057525	0.07263871
810	2575	7.853604813	106.4088433	0.073805941	0.052199691
811	2569	7.851271997	98.37378718	0.07981061	0.05643967
812	1226	7.111512116	61.67424942	0.115307639	0.078091606
813	1343	7.202661197	61.67424942	0.116785551	0.07955922
814	893	6.794586581	47.42032316	0.143284274	0.094938818
815	67	4.204692619	42.26728324	0.099478658	0.04798388
816	22009	9.99920674	106.4088433	0.093969697	0.0723345
817	5298	8.57508467	100.5149849	0.085311505	0.062420513
818	477	6.167516491	99.35880992	0.062073172	0.039086856
819	475	6.163314804	93.98401306	0.065578332	0.041278295
820	4821	8.480736654	99.35880992	0.085354652	0.06219898
821	1691	7.433075349	97.87357084	0.075945685	0.052473803
822	3130	8.048788284	97.87357084	0.082236586	0.058739805
823	2739	7.915348169	94.91358957	0.083395309	0.059170068
824	1351	7.208600338	89.77772984	0.080293859	0.054720195
825	1388	7.235619141	89.77772984	0.080594811	0.055019182
826	1142	7.04053639	87.05739757	0.080872351	0.054513476
827	16711	9.723822464	100.5149849	0.096740028	0.073837506
828	6789	8.823058934	97.67782763	0.090328165	0.066768465
829	6779	8.821584877	84.33848877	0.104597379	0.077311399

830	9922	9.202509793	97.67782763	0.094212883	0.070648903
831	3089	8.035602693	86.23435909	0.093183306	0.066515563
832	2272	7.72841578	79.43999726	0.097286204	0.068350759
833	817	6.705639095	79.43999726	0.084411371	0.055564071
834	808	6.694562059	64.9310724	0.103102595	0.067811201
835	257	5.549076085	52.43271478	0.105832324	0.062573743
836	551	6.311734809	52.43271478	0.120377799	0.076771758
837	6833	8.829519095	86.23435909	0.102389804	0.075703587
838	316	5.755742214	80.99875898	0.071059635	0.042978931
839	222	5.402677382	75.07424339	0.07196446	0.041823034
840	94	4.543294782	75.07424339	0.060517357	0.031064501
841	6517	8.782169426	80.99875898	0.108423506	0.080013132
842	38	3.63758616	75.97879268	0.047876335	0.020368348
843	6479	8.776321456	75.97879268	0.115510146	0.085222787
844	5446	8.602636673	72.31582925	0.118959248	0.087141403
845	5400	8.594154233	65.71780396	0.130773606	0.095761484
846	5197	8.555836815	59.30228081	0.144275004	0.105476247
847	2997	8.005367067	57.66459364	0.138826385	0.098947727
848	2200	7.696212639	57.66459364	0.133465133	0.093605274
849	2198	7.695303135	55.58551727	0.138440794	0.097090116
