# Supplementary material for: A Complete Fossil-Calibrated Phylogeny of Seed Plant Families as a Tool for Comparative Analyses: Testing the ‘Time for Speciation’ Hypothesis
Source: PLoS One. 2016 Oct 5;11(10):e0162907. doi: 10.1371/journal.pone.0162907 (PMC5051821; doi:10.1371/journal.pone.0162907)
Supplement: S5 Table — (PDF) [file pone.0162907.s007.pdf]

Table S5: Model parameters for the MEDUSA model. These models are mapped to the phylogeny in Fig 3.

Model Number	Diversification Rate (r)	Extinction rate (ε)	Lower Bound (r)	Upper Bound (r)	Lower Bound (ε)	Upper Bound (ε)
1	0.006	0.993	0.004	0.008	0.990	1.000
2	0.050	0.974	0.048	0.053	0.971	1.000
3	0.537	NA	0.458	0.690	NA	NA
4	0.256	NA	0.216	0.334	NA	NA
5	0.038	NA	0.025	0.056	NA	NA
6	0.384	NA	0.317	0.513	NA	NA
7	0.438	NA	0.360	0.590	NA	NA
8	0.300	NA	0.250	0.397	NA	NA
9	0.262	NA	0.230	0.307	NA	NA
10	0.270	NA	0.223	0.360	NA	NA
11	0.153	NA	0.136	0.178	NA	NA
12	0.237	NA	0.196	0.315	NA	NA
13	0.228	NA	0.211	0.249	NA	NA
14	0.035	0.826	0.027	0.045	0.696	1.000
15	0.021	NA	0.000	0.046	NA	NA
16	0.031	NA	0.020	0.049	NA	NA
17	0.325	NA	0.285	0.382	NA	NA
18	0.088	0.987	0.076	0.104	0.978	1.000
19	0.057	NA	0.034	0.092	NA	NA
20	0.221	NA	0.195	0.257	NA	NA
21	0.219	NA	0.187	0.271	NA	NA
22	0.065	NA	0.052	0.082	NA	NA
23	0.015	NA	0.000	0.041	NA	NA
24	0.038	NA	0.000	0.069	NA	NA
25	0.000	NA	0.000	0.016	NA	NA
26	0.005	NA	0.000	0.022	NA	NA
27	0.011	NA	0.000	0.048	NA	NA
28	0.000	NA	0.000	0.035	NA	NA
29	0.161	NA	0.134	0.212	NA	NA
30	0.162	NA	0.142	0.191	NA	NA
31	0.005	NA	0.000	0.024	NA	NA
32	0.009	NA	0.000	0.027	NA	NA
33	0.024	NA	0.000	0.053	NA	NA
34	0.406	NA	0.337	0.538	NA	NA
35	0.308	NA	0.274	0.355	NA	NA
36	0.000	NA	0.000	0.020	NA	NA
37	0.023	NA	0.000	0.063	NA	NA
38	0.006	NA	0.000	0.029	NA	NA
39	0.156	NA	0.130	0.206	NA	NA
40	0.011	NA	0.000	0.033	NA	NA
41	0.039	NA	0.027	0.057	NA	NA
42	0.000	NA	0.000	0.027	NA	NA
43	0.000	NA	0.000	0.027	NA	NA
44	0.000	NA	0.000	0.028	NA	NA
45	0.033	NA	0.000	0.073	NA	NA
46	0.179	NA	0.129	0.276	NA	NA
47	0.007	NA	0.000	0.035	NA	NA

48	0.000	NA	0.000	0.032	NA	NA
49	0.021	0.997	0.016	0.027	0.996	1.000
